# The host-range, genomics and proteomics of *Escherichia coli* O157:H7 bacteriophage rV5

**DOI:** 10.1186/1743-422X-10-76

**Published:** 2013-03-06

**Authors:** Andrew M Kropinski, Tom Waddell, Juncai Meng, Kristyn Franklin, Hans-Wolfgang Ackermann, Rafiq Ahmed, Amanda Mazzocco, John Yates, Erika J Lingohr, Roger P Johnson

**Affiliations:** 1 Public Health Agency of Canada, Laboratory for Foodborne Diseases, 110 Stone Road West, Guelph, ON N1G 3W4, Canada; 2 Department of Molecular and Cellular Biology, University of Guelph, Guelph, ON, N1G 2W1, Canada; 3 Abbott Point of Care, 185 Corkstown Road, Ottawa, ON, K2H 8V4, Canada; 4 Merck Research Laboratories, 126E Lincoln Avenue, Rahway, NJ, 07065, USA; 5 Département de Microbiologie-infectiologie et immunologie, Faculté de médecine, Université Laval, Québec, QC, G1K 7P4, Canada; 6 Enteric Diseases Program, National Microbiology Laboratory, Public Health Agency of Canada, 1015 Arlington Street, Winnipeg, MB, R3E 3R2, Canada; 7 The Scripps Research Institute, Department of Cell Biology, Proteomic Mass Spectrometry Laboratory, 10550 North Torrey Pines Road, La Jolla, CA, 92037, USA

**Keywords:** *E. coli* O157:H7, Phage evolution, Phage ecology, *Myoviridae*, Genomics, Proteomics, Bioinformatics, Pyrosequencing, Tail spike

## Abstract

**Background:**

Bacteriophages (phages) have been used extensively as analytical tools to type bacterial cultures and recently for control of zoonotic foodborne pathogens in foods and in animal reservoirs.

**Methods:**

We examined the host range, morphology, genome and proteome of the lytic *E. coli* O157 phage rV5, derived from phage V5, which is a member of an *Escherichia coli* O157:H7 phage typing set.

**Results:**

Phage rV5 is a member of the *Myoviridae* family possessing an icosahedral head of 91 nm between opposite apices. The extended tail measures 121 x 17 nm and has a sheath of 44 x 20 nm and a 7 nm-wide core in the contracted state. It possesses a 137,947 bp genome (43.6 mol%GC) which encodes 233 ORFs and six tRNAs. Until recently this virus appeared to be phylogenetically isolated with almost 70% of its gene products ORFans. rV5 is closely related to coliphages Delta and vB-EcoM-FY3, and more distantly related to *Salmonella* phages PVP-SE1 and SSE-121, *Cronobacter sakazakii* phage vB_CsaM_GAP31, and coliphages phAPEC8 and phi92. A complete shotgun proteomic analysis was carried out on rV5, extending what had been gleaned from the genomic analyses. Host range studies revealed that rV5 is active against several other *E. coli.*

## Background

Since *Escherichia coli* O157:H7 is associated with foodborne illness in humans with serious complications such as hemorrhagic colitis and the hemolytic uremic syndrome, much effort has been directed at understanding the epidemiology and virulence of this zoonotic bacterium [1,2], and minimizing its carriage by cattle through phage biocontrol [3-5].

The scientific literature lists over fifty phages described as being *E.coli* O157-specific. These include sixteen phages (V1-V16) comprising part of a phage typing scheme for this bacterium [6] plus phages 38, 39, 41, 42, ECB7 and ECA1 [7]; AR1 [8,9]; Bo-21, Av-05, Av-06, and Av-08 [10]; CA933P, CA911 MFA933P and MFA45D [11]; CEV1 and CEV2 [12,13]; CSLO157 [14]; DC22 [15], e4/1c and e11/2 [16]; ECML-4, ECML-117, and ECML-134 [17]; JK06; KH1, KH4 and KH5 [18]; LG1 [19]; φV10 [20,21]; PP01 [22]; SFP10 [23]; SH1 [24]; SP15, SP21, and SP22 [25]; vB_EcoM_CBA120 [5]; vB_EcoS_AKFV33 [4]; and, vB_EcoS_Rogue1 [26]. However, relatively little or consistent information on morphology and taxonomic position, host range, receptor specificity, genome size and characterization is available for many of these viruses.

Only a limited number of these viruses have been fully sequenced. They include members of the *Myoviridae* (AR1, V7, wV8, CBA120, SFP10), *Siphoviridae* (JK06; Rogue1; AKVF33) and *Podoviridae* (φV10) viral families. All are lytic phages except the latter virus which is temperate. The myoviruses include representatives of three viral genera: the “FelixO1likevirus” (wV8; [27,28]), the “Viunalikeviruses” (CBA120 and SFP10; [29] and the “T4likeviruses” (AR1 and V7 [30]) and the “T5like viruses” (AKVF33). The siphoviruses belong to the “Tunalikevirus” genus (JK06, Rogue1) or “T5likevirus” (AKFV33), while the member of the *Podoviridae* is related to Group E1 *Salmonella enterica*-specific bacteriophage ε15 [31], making it a member of the “Epsilon15likevirus” genus [28].

We describe here the host range, morphology, genome and proteome of a phage designated rV5, considered a derivative of the typing phage V5 of the original *E. coli* O157:H7 phage typing set [6]. Phage rV5 was the predominant phage recovered (hence “r”V5) from the feces of calves experimentally infected with *E. coli* O157:H7 and treated successfully with a cocktail of six of these typing phages including V5 during a phage therapy trial [32,33]. Although having the same host range as V5, as shown below, rV5 was considered distinct from V5 as rV5 may have acquired other attributes during passage through the calves that would enhance its value as a candidate therapeutic phage.

## Results

### Host-range of phage rV5

The phage was tested for lytic activity on reference strains of 12 common phage types of *E. coli* O157:H7 and the ECOR collection [34]. The host range and activity of rV5 on these 12 is the same as previously found for phage V5 (data not shown). Six (50%) of the 12 O157:H7 phage type reference strains were susceptible; four being highly susceptible (>50% lysis) (Additional file 1: Table S1). Seventeen (24%) of 72 strains of the ECOR collection showed evidence of lysis, although only one strain was highly susceptible (>50% lysis) (Additional file 2: Table S2) Among these 17 strains, five had O antigens shared by other diarrheagenic E. coli: O7, enteroaggregative *E. coli*; O25 and O173, enterotoxigenic *E. coli;* O113, enterohemorrhagic *E. coli*; and O167, enteroinvasive *E. coli*[35].

### Morphology of rV5

Phage rV5 has a contractile tail and is therefore a member of the *Myoviridae* family. This virus has an icosahedral head with a diameter of 91 nm between opposite apices. The extended tail measures 121 × 17 nm and has a sheath of 44 × 20 nm and a 7 nm-wide core in the contracted state. Five to six thin tail fibers of 70 nm in length are occasionally seen (data not shown).

### Properties of the phage genome

**Figure 1:**
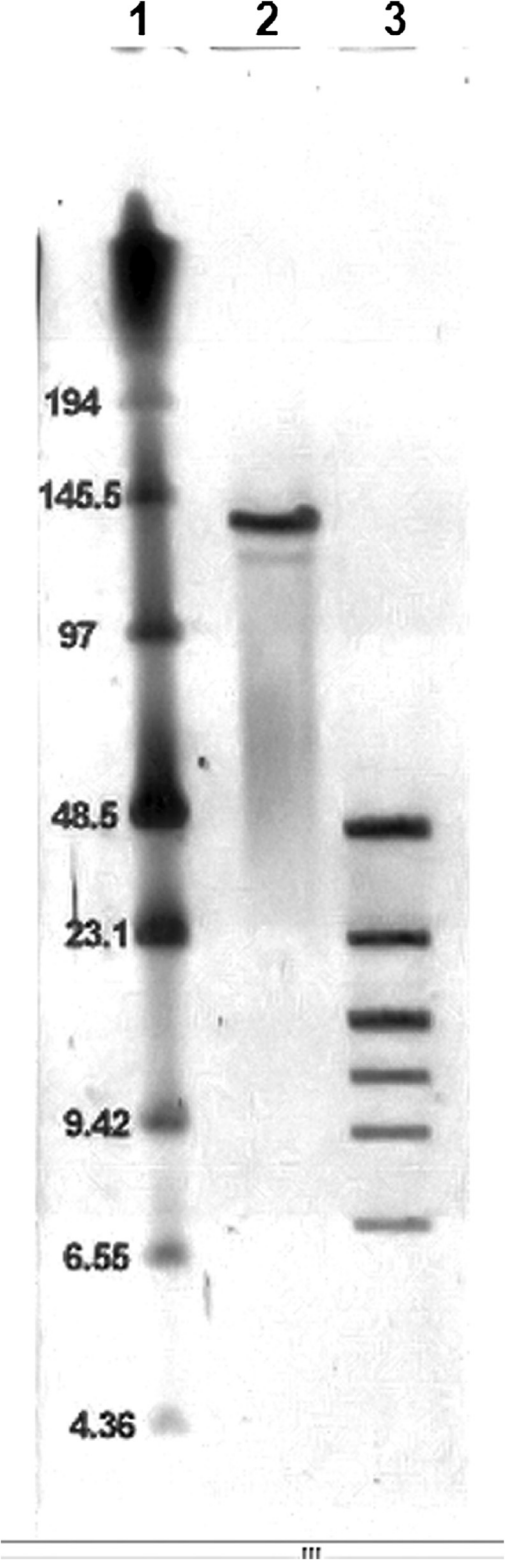
**PFGE analysis of rV5 DNA. PFGE was performed on rV5 genomic DNA with and without digestion with XbaI. **Lane 1, NEB Low Range PFGE Marker; lane 2, rV5 genomic DNA, Lane 3, rV5 genomic DNA digested with XbaI.

The sequence of the rV5 phage genome was determined through sequencing of two random clone libraries and by primer walking using the phage DNA as a template. All 846 sequence reactions at approximately 600 bp per reaction resulted in 3.6 fold coverage of the genome. The final sequence of the circularly permuted genome (137,947 bp, 43.6 mol% GC) is very similar to the size estimated by PFGE (132.5 kb; Figure 1). An analysis of the variation in base composition over the entire length of the genome revealed very little evidence of horizontally acquired genes [36].

### tRNAs

Like many of the larger members of the *Myoviridae*, rV5 codes for tRNAs. Five (Arg_AGA_, Tyr_TAC_, Thr_ACA_, Met_ATG_, Pro_CCA_) were identified using the tRNAScan program [37] and an additional one (Ser_TGA_) was detected using ARAGORN [38]. In *E. coli* O157:H7 strains AGA is used as the Arg codon 5.1% of the time, followed by threonyl codon ACA (14.6%), prolyl codon CCA (19.1%), tyrosyl codon TAC (42.7%), and methionyl codon ATG (100%). By comparison, rV5 uses these same codons 26, 35, 29, 46 and 100% of the time. It would appear that the presence of the tRNA_Arg_ and the tRNA_Pro_ homologs would increase the rate of translation of phage mRNAs. Methionyl tRNA, while seemingly unwarranted, occurs in many members of the *Myoviridae* including *Aeromonas* phage Aeh1 (2 copies, NC_005260), mycobacteriophages Bxz1 (2 copies, NC_004687), vibriophage KVP40 (NC_005083), *Listeria* phage P100 (NC_007610), and *Synechococcus* phage S-PM2 (3 copies, NC_006820). This suggests that the presence of additional tRNA_Met_ may facilitate the rapid translation of phage mRNAs.

### Identification of ORFs

**Figure 2:**
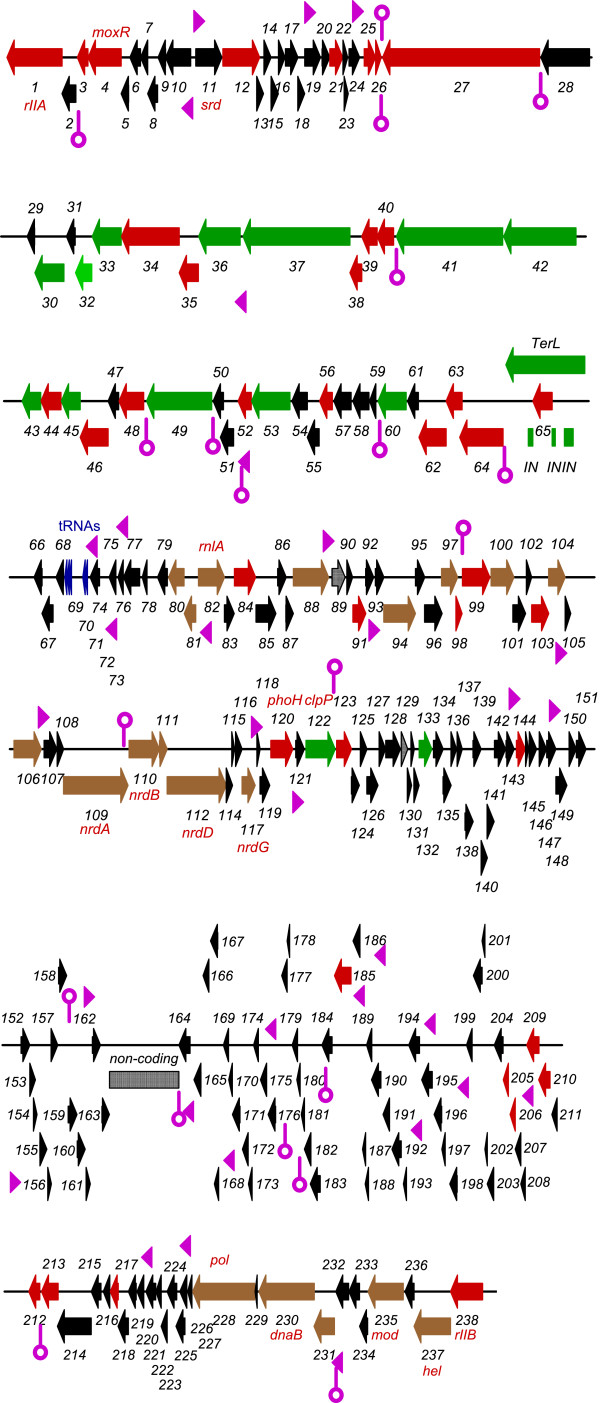
**Genetic map of phage rV5 with each line representing 21 kb of the sequence. **Genes in colour represent those whose products exhibit homologs in the NCBI nonredundant databases, while those illustrated in black lack homologs. Green, brown and grey colored genes specify proteins involved in morphogenesis, DNA metabolism and lysis, respectively. The grey box labeled “non-coding” contained no ORFs. Promoters are illustrated as pink arrowheads, while rho-independent terminators are displayed as stem-loop structure, also in pink.

The ORFs for rV5 were identified using the Kodon software package from Applied Maths (Austin, TX). In almost every case upstream there was a sequence showing considerable similarity to the consensus ribosome-binding site (5^′^GGAGGT3^′^). A total of 233 ORFs were discovered most closely packed or overlapping. The total codon capacity of the genome was 91.6% (average 0.54 kb per ORF) (Figure 2). The rV5 genome contained 88 mainly small ORFs between 92269–121323 and no observable ORFs from regions 104013–106618. Prior to our description of *Salmonella* phage PVP-SE1 [39], only 73 (31%) of gene products of rV5 possessed homologs to proteins in the nonredundant databases; and, only 44 (19%) were homologous to phage proteins. The rV5 proteome was scanned with TMHMM [40], and Phobius [41] programs, revealing that 15 proteins possessed transmembrane domains (Additional file 3: Table S3).

### Transcription

From the gene layout in Figure 2, we propose that rV5 contains four transcriptional units comprising genes *10-1-238-164, 11–26, 27–81*, and *82*–*163*, respectively. Based upon the gene arrangement, we would minimally expect bidirectional transcriptional terminators between genes *26* and *27* and genes *163* and *164*, and bidirectional promoters between genes *10* and *11* and *81* and *82*, respectively. Of these sites, only the bidirectional terminators were located between genes *26* and *27*. In addition, bidirectional promoters were discovered between genes *10* and *11*. In total, using stringent selection processes, 33 promoters and 20 rho-independent terminators where discovered in the rV5 genome (Additional file 4: Table S4). All had extensive homology to the consensus *E. coli* promoters, with 11 possessing extended -10 regions [34,35]. Since these promoters are distributed across the rV5 genome, it suggests that modification of the host holo-RNA polymerase, as observed with coliphage T4 to permit recognition of different promoter classes [42], might not occur in rV5. To investigate this further, we selected the upstream sequence for late genes (*27*–*66*) and resubmitted it to MEME [43]. Eight copies of a motif (TggTAaAAtA) which is similar to the T4 late promoter consensus sequence (TATAAATA) [44,45], were identified (Additional file 4: Table S4). Late transcription in T4-like phages is dependent upon three gene products, namely gp45 (RNA polymerase recruitment), gp33 (co-activator of late transcription) and gp55 (late promoter recognition protein). There are no homologs for these proteins in rV5.

PSI-BLAST analysis of gp11 revealed that it is probably a Srd homolog. These proteins are postulated to act as antisigma factors functioning as decoys for RpoD and RpoS. It is homologous to similar proteins in coliphages T4 (NP_049634), *Acinetobacter* phage 133 (YP_004300600) and *Pseudomonas* phage φPto-bp6g (AEO14611). Perhaps this is used as a part of a molecular switch between early and late transcription.

### Nucleotide metabolism and DNA replication

Phage rV5 contains numerous genes involved in nucleotide metabolism and DNA replication. Among the former we found genes coding for exo- (gp94) and endodeoxyribonucleases (gp*213*), the anaerobic and aerobic ribonucleotide reductase subunits (gp109-112 and *117*), and thymidylate synthase (gp106). This group of enzymes is also commonly found in many other members of the *Myoviridae* and is collectively responsible for generating deoxyribonucleotides for phage DNA synthesis. The ribonucleoside-diphosphate reductases are responsible for the interconversion of ribo- to deoxyribonucleotides and are usually represented by three main classes: class I complex of NrdAB or NrdEF which requires oxygen for activity; class II containing NrdJ and the oxygen-sensitive; class III encoded by NrdDG [46]. As with coliphages RB43, RB49 and RB69, phage rV5 contains homologs of the hosts NrdAB and NrdDG proteins.

Among the enzymes directly involved in DNA replication are a DNA ligase (gp88), DNA polymerase (gp228), and two possible helicases (gp230, gp237). gp88 contains a PRK09125 DNA ligase domain and its closest homolog in ATP-dependent DNA ligase of Enterobacteria phage vB_EcoM-FV3 (AEZ65217), *Salmonella* phage 7–11 (YP_004782418) [47] and *Pseudomonas* phage P3_CHA (ADX32167) [48]. The 775 amino acid rV5 DNA polymerase (gp*228*), possesses a smart00482 (POLAc) DNA polymerase A and, a DNA_pol_A_pol_I_B (cd08643) domain. Its closest homologs are in Enterobacteria phage vB_EcoM-FV3 (AEZ65345), *Cronobacter* phage CR3 (AFH21225) [49] and *Vibrio* phage ICP1_2001_A (ADX89239) – all members of the *Myoviridae*. Gp*230* contains C-terminal GP4d_helicase (cd01122) and DnaB (COG0305) domains. Again its homologs are to proteins in vB_EcoM-FV3 (AEZ65346), CR3 (AFH21242) as well as to primase/helicases in members of the *Podoviridae.* The product of gene *237* has PIF1 (pfam05970), PIF1-like helicase and RecD (COG0507), ATP-dependent exoDNAse (exonuclease V), alpha subunit domains, and again shows homology to proteins in phages vB_EcoM-FV3 and CR3.

In an effort to define the origin of replication of this phage, Grigoriev AT- and GC-skew analysis was undertaken [50-53]. The rV5 genome revealed changes at nucleotides 6425, 13675–13725, 66675–66725 and 104425–105475, all of which appear to be associated with a change in the orientation of transcription.

### Proteomics and morphogenesis

The proteomics of rV5 were investigated in three ways. (1) The proteins were screened for homologs to structural proteins in other phages using the BLASTP program, (2) the virions were studied by one-dimensional SDS-PAGE (data not shown) and (3) the total phage proteome was investigated by mass spectrometry (Additional file 5: Table S5). SDS-PAGE revealed at least 10 bands, the five major ones having relative molecular weights of 288.2, 174.0, 52.3, 26.1 and 9.7 kDa. Among the proteins detected by total phage proteomics were the putative tail proteins (gp37, 42 and 49), tail fibre proteins (gp30, 32, 33, 41 and 43), tail baseplate (gp36 and 45), and a major capsid protein (gp60).

The five proteins that deserve further attention are gp30, 33, 37, 41 and 43 since they appear to specify tail fiber-like proteins which play crucial roles in phage adsorption to its host. These proteins were analyzed using HHpred [54]. Gp30, a 347 amino acid protein, contained a domain with significant similarity (Probab=98.39 E-value=9e-08) to the short tail fibers of coliphage T4 (Gp12) which are involved in LPS-binding (PDB accession number 1PDI; [55]). Interestingly, the similarly sized Gp*33* also shows significant homology (Probab=97.69 E-value=7.5e-06) to this same protein. These two proteins show 42.3% sequence identity using the ALIGN Query program [56] which suggests that two chemotypes of LPS may be recognized.

With 1279 amino acid residues, gp37 is one of the largest proteins specified by this virus. Its domains include COG4733 [phage-related protein, tail component]. The phage homologs include *Shewanella* prophage MUSO2, 43 kDa tail protein 3CDD (Probab=97.13 E-value=0.011) and a *Neisseria* 43 kDa prophage tail protein (Probab=97.05 E-value=0.0095). Gp41, a 1272 AA protein, possesses a C-terminal domain (3GW6, Probab=98.69 E-value=1.5e-08) to an endo-N-acetylneuraminidase from Enterobacteria phage K1F, a podovirus. This region shows a high probability of a coiled-coil structure as demonstrated using PCOILS [57,58]. The N-terminus of gp43 (222 AA) shows structural similarity to the N-terminus of phage P22 tailspike protein (2VNL; Probab=96.34 E-value=0.00042).

Using Using mass spectrometry of trypsin-digested virions the following proteins were identified: gp52 (tail tube protein; 16.1% coverage), gp53 (tail sheath protein; 31.9%), gp60 (major capsid protein; 83.3%), gp61 (head decoration protein; 85.3%), gp64 (portal protein; 36.3%) all of which are expected to be major components of the viral particles. In addition, gp133 (15.9%) was one of the predominant proteins (Additional file 5: Table S5). A comparison of phage rV5 and phi92 [59] permitted us to definitively identify the tail tube and sheath proteins.

### Introns in terminase

BLASTX analysis revealed that the gene specifying the large subunit of the terminase complex was divided into three segments, one of which contained a homing endonuclease. While introns are not uncommon in myoviral genomes, being present in coliphage T4 [42], *Aeromonas* phage 25 (NC_008208), *Pseudomonas* phage φEL, and *Synechococcus* phage S-PM2, in only one other virus, siphovirus LL-H of *Lactobacillus delbrueckii* subsp. *Lactis*, does the TerL gene contain an intron [60].

### Lysis

Lysis of infected bacteria is brought about through the sequential effects of a pore-producing protein – the holin – and a peptidoglycan-degrading enzyme – the lysin. Holins usually contain 2–3 membrane spanning helices (TMD), a charged C-terminus and exhibit poor sequence identity to other functionally related proteins [61-63]. In many phages, a lysis cassette exists in the genome with the holin gene preceding that of the lysin. In rV5, Gp*89* codes for an obvious lysin (pfam00959, Phage_lysozyme & COG467, Muramidase) possessing strong sequence identity to the lysozymes of enterobacterial phages phage vB_EcoM-FV3, and *Salmonella* phage Vi II variant E1 [64]. Since no homolog to a holin was discovered, the rV5 proteome was scanned with TMHMM [40] and Phobius [41]. In only one case, gp129*,* did the two programs indicate that the protein contained two TMDs. This 78 amino-acid residue protein also possessed a high concentration of lysyl- and arginyl-residues in its C-terminus suggesting that this putative holin is separated from to the lysin gene as in phage T4.

## Discussion

### Host range studies

Phage rV5 was subject to extensive host range studies, revealing virulence for numerous *E. coli* other than serotype O157:H7. The six *E. coli* O157:H7 phage type reference strains susceptible to rV5 together represent 73% of all isolates of *E. coli* O157:H7 phage typed at the National Microbiology Laboratory in Canada in 2007–2010 [65] [The National Microbiology Laboratory (NML) and Centre for Food-borne Environmental and Zoonotic Infectious Diseases (CFEZID) PHAC, Provincial Public Health Microbiology Laboratories. 2010 Annual Summary of Laboratory Surveillance Data. Forthcoming]. Also, among the susceptible *E. coli* strains of the ECOR collection were several that share the same O antigens as other diarrheagenic *E. coli.* Since O antigens are recognized as attachment sites for phages of Gram-negative bacteria, rV5 potentially may be activity against diarrheagenic *E. coli* other than *E. coli* O157:H7. Virulence for such a broad range of pathogens potentially is of value for candidate therapeutic phages, as has been noted previously [66].

### Evolutionary considerations

The phylogenic origin of specific phages is always complicated by recombinational exchanges that have presumably occurred during the speciation of the virus. When this study was initiated in 2004, phage rV5 was a genomic orphan since the majority (ca. 70%) of its genes were ORFans [67,68]. Since then five other phages have been reported to be rV5-like: coliphages vB_EcoM-FV3 [69], phAPEC8 [70] and phi92 [59], *Cronobacter sakazakii* phage vB_CsaM_GAP31 [71] and *Salmonella* phage PVP-SE1 [39]. To this list we can also add *Salmonella* phage SSE-121 (JX181824); and, coliphage Delta Y that Andrey Letarov and Alla Golomidova (Winogradsky Institute of Microbiology, RAS, Moscow, Russia), isolated from horse manure, and partially sequenced. This once again illustrates that very similar phages may be isolated from widely different locales [72-74].

**Figure 3:**
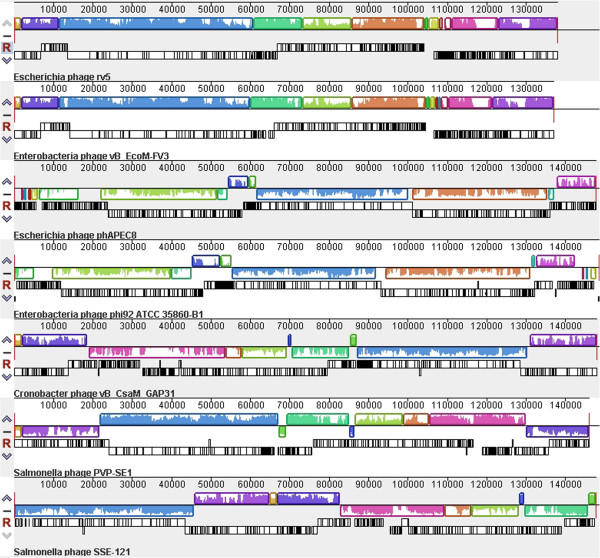
**ProgressiveMauve alignment of seven phage genomes which are related to coliphage rV5. **The blocks of similar colour for each phage indicate regions of DNA sequence relatedness; while white regions indicate dissimilar sequence. Below these are illustrated the phage genes as black outlined boxes on the plus (above horizontal) and minus (below horizontal) strands. Please note that the genomes of rV5 and FV3; and, phAPEC8 and phi92 are collinear with each other; and that the initial brown segment in rV5 is found in the same position in FV3, GAP32 and PVP-SE1 (but in this case of the complementary strand); at ca. 65kb in the sequence of SSE-121; and is entirely missing is phi92 and phAPEC8. The fact that many of these genomes are not collinear renders direct comparisons difficult.

**Figure 4:**
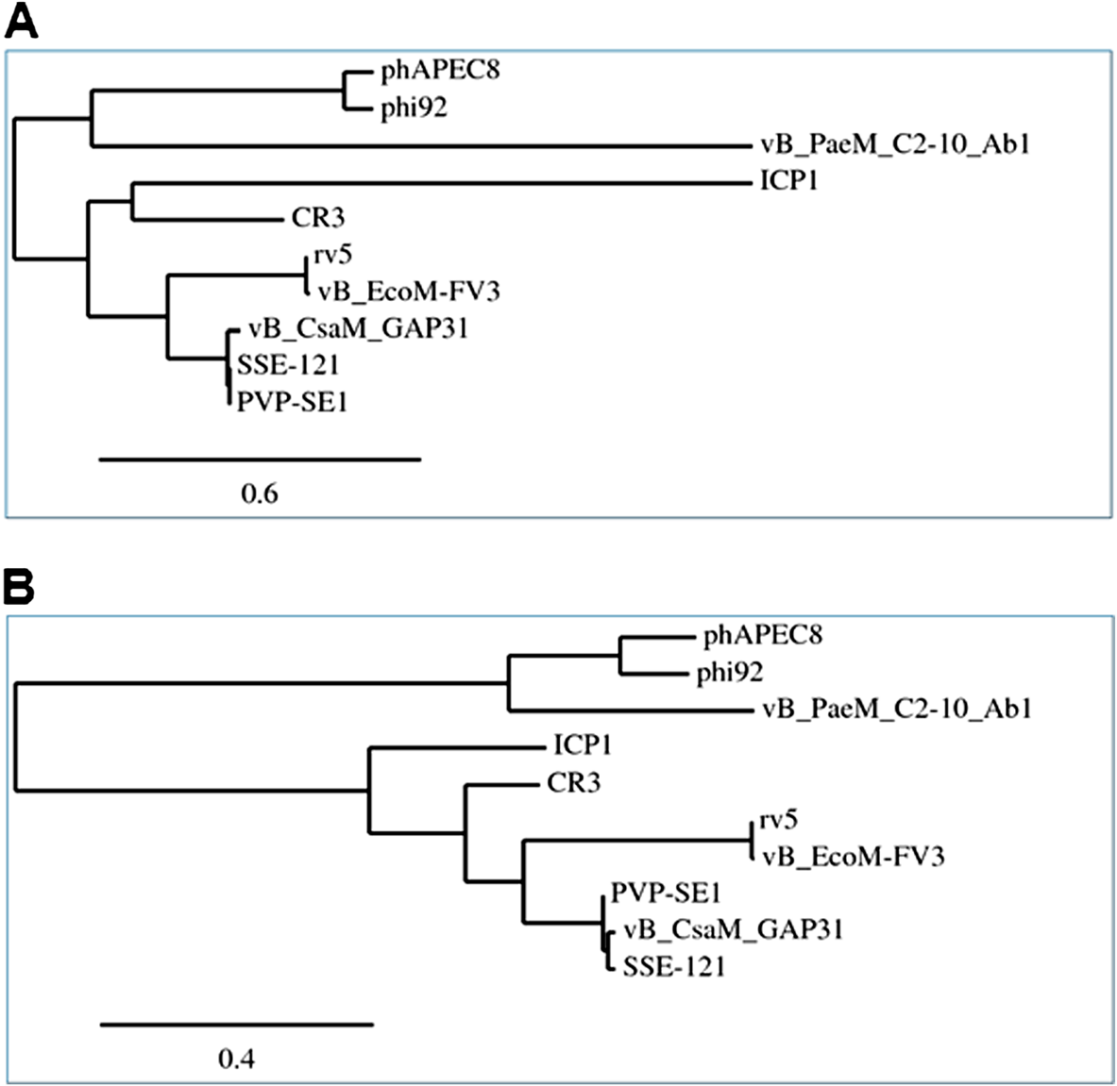
**Phylogenetic analysis of rV5-related phage capsids protein (A) and DNA polymerases (B) using “one click at phylogeny.fr.** Homologous proteins from *Cronobacter* phage C3 (NC_017974), *Vibrio *phage ICP1 (NC_015157) and *Pseudomonas *phage vB_PaeM_C2-10_Ab1 (HE983845) were used as outliers.

Based upon the proposed assignment to a genus being the presence of 40% conserved proteins [28,75], the five fully sequenced phages could be grouped in the “V5likevirus” genus. The submitting author is now of the opinion that the use of the 40% protein homologs as an indication of membership in the same genus is too inclusive, resulting in, at least for the phages with large proteomes, “taxonomic lumping.” At the protein level, rV5 and FV3 share 90.6% homologous proteins; while rV5 and PVP-SE1, only share 42.9% of the proteomic content. At the DNA level, rV5 and coliphage vB_EcoM-FV3 share 87.3% DNA sequence identity, while rV5 and *Salmonella* phage PVP-SE1 share <50% sequence identity. Based upon BLASTN analysis the mycobacteriophages have been grouped and subgrouped (http://phagesdb.org/; [76]). Using the same approach, complemented by progressiveMauve analyses (Figure 3) [77] we visualize the existence of three related genera - the “V5likevirus” (rV5, FV3), the “Pvplikevirus” (PVP-SE1, GAP31 and SSE-121) and the Phi92likevirus (phi92 and phAPEC8). The results of the progressiveMauve alignment also indicate a serious problem with the genomics of phages with circularly permuted genomes, that the genomes are not collinear. This is most apparent with the “Pvplikevirus” all of which start in radically different positions, which require realignment before running EMBOSS stretcher. The separation of the rV5-related phages into three groups is also indicated by a phylogenetic analysis of their capsid proteins and DNA polymerases which clearly indicate three clades (Figure 4).

## Materials and methods

### Bacteriophages and hosts

Phage V5 was obtained from Rafiq Ahmed (National Microbiology Laboratory, Winnipeg, MN, Canada) and is part of a collection of E.coli O157:H7 typing phages [6]. Phage rV5 was isolated during a successful “proof of concept” study of phage therapy for *E. coli* O157:H7 infection of cattle; it was the predominant phage in the feces of calves that eliminated *E. coli* O157:H7 following oral administration of a mixture of V5 and five other lytic O157 phages [32,33]. Determination of the host range of rV5 and V5 propagated and quantitated on *E. coli* O157:H7 strain R508 for 12 *E. coli* O157:H7 phage type reference strains revealed they shared the same host range, consistent with the designation of rV5 as a derivative of V5.

### Host range study

The virulence of phage rV5 for reference strains of 12 common phage types of *E. coli* O157:H7 and 72 strains of the ECOR collection [34] was determined by spotting 10^5^ PFU of phage rV5 onto freshly seeded lawns of bacteria on agar plates [6].

### Electron microscopy

Phage rV5 was sedimented for 60 min at 25,000 g in a Beckman J2-21 ultracentrifuge (Palo Alto, CA) using a JA-18.1 fixed angle rotor, and washed twice in buffer (0.1 M neutral ammonium acetate). The sediment was deposited on carbon-coated copper grids, stained with 2% potassium phosphotungstate (pH 7.0) and 2% uranyl acetate (pH 4.0), and then examined in a Philips EM 300 electron microscope operated at 60 kV. Magnification was monitored using T4 phage tails (113 nm in length) [78]. Particles were measured on prints at a final magnification of 297,000 times.

### Propagation of phages and their purification

The phages were propagated at a multiplicity of infection (MOI) of 10 on *E. coli* O157:H7 strain R508 in 2.0 L of TSB containing 10 mM MgSO_4_ for 18 h at 37°C with shaking at 120 rpm. The resulting lysates were clarified by centrifugation at 6,000 × *g* and pancreatic DNase 1 and RNase A (Sigma Aldrich, St. Louis, MO) were added to the filtrate to concentrations of 10 μg/ml. The phages were precipitated with polyethylene glycol 8000 [79], and subsequently purified by cesium chloride step and equilibrium density gradient ultracentrifugation as described by Sambrook and Russell [80]. The final band was dialyzed at 4°C against two changes of 2 L of dialysis buffer (10 mM Tris HCl, 10 mM MgSO_4_.7H_2_0, 25mM NaCl, pH 7.5, 0.01% gelatin). The concentration of purified phages in the dialyzed suspension was determined by direct plaque assay with *E. coli* O157:H7 strain EC990298 as the host.

### Pulsed field gel electrophoresis (PFGE)

The genome size of rV5 was characterized by PFGE [81] and data were analyzed using the BioNumerics program (Applied Maths, Austin, TX).

### Purification of phage DNA

DNA for construction of a clone library was extracted from phage rV5 prepared as above to the stage of precipitation with PEG 8000 and sedimentation by ultracentrifugation. The pellet was resuspended in a minimal volume of lambda diluent. EDTA was then added to a concentration of 20 mM, and the phage DNA was extracted by sequential treatment with proteinase K (50 mg/ml), SDS (0.5%, w/v), phenol-chloroform extraction and ethanol precipitation [80]. The precipitated DNA was dissolved in water, tested for purity by electrophoresis in 0.9% agarose and by PCR for contaminating bacterial DNA using the *malM* gene of *E. coli* O157:H7 as a target. The concentration of DNA in the final preparation was calculated from its absorbance at 260 nm.

### Genome sequencing

The sequence of rV5 was derived initially from a clone library and subsequently by primer walking at The Centre for Applied Genomics (Toronto, ON, Canada). Primers were designed using Premier Biosoft’s NetPrimer (http://www.premierbiosoft.com/netprimer/), and purchased from Sigma Genosys Canada (Oakville, ON). The sequences were assembled using the SeqMan program (DNASTAR, Madison, WI).

### Genome annotation

Open reading frames (ORFs) were identified using Kodon (Applied Maths). The protein products of each ORF were examined for homologs using the programs PSI-BLASTP [82,83] or Batch-BLAST (http://greengene.uml.edu/programs/NCBI_Blast.html. In certain cases the proteins were also subjected to HHpred [54,84] analysis at http://toolkit.tuebingen.mpg.de/hhrep. In addition, each protein was scanned for conserved protein motifs using Batch Web CD-Search Tool [85,86], TMHMM [41] and Phobius [41]. Transfer RNAs were detected using tRNAscan [37] and ARAGORN [38]. Codon usage information on *E.coli* O157:H7 strains was determined using data from the Forsyth Institute’s Microbial Genome Codon Usage Database (http://exon.gatech.edu/metagenome/CodonUsageDatabase/). The codon usage of rV5 was analyzed using DNAMAN software (Lynnon Corp., Vaudreuil-Dorion, QC, Canada). Potential terminators were located by ARNold [87] and verified using the MFOLD algorithm [88]. Putative promoters were identified in the sequence upstream (5^′^) of the genes by homology to the consensus sigma-70 promoters of *E. coli* (TTGACA (N15-20) TATAAT) using the “search sequences” feature of DNAMAN. As a further aid to identifying interesting regulatory sequences 100 bp of 5^′^ upstream sequence data was extracted using extractUpStreamDNA at http://lfz.corefacility.ca/extractUpStreamDNA/ extractUpStreamDNA/ and submitted to MEME [89] at http://meme.sdsc.edu/.

For comparative genomic analyses we employed EMBOSS Stretcher at http://emboss.bioinformatics.nl/cgi-bin/emboss/stretcher, while CoreGenes 2.0 [90,91] was used to compare proteomes. Phylogenetic analyses were carried out using “one click” at http://www.phylogeny.fr/[92].

### Genome accession numbers

The annotated genomic sequence of phage rV5 is available from the NCBI under the accession number DQ832317.

### Proteomics (sample preparation and MudPIT analysis)

After unsuccessful attempts to disrupt phage rV5 by osmotic shock with sodium chloride, it was treated with LiCl (2). Six ml of 10 M LiCl were added to 6 ml of purified dialysed phage rV5 containing 1.2 × 10^12^ PFU. The mixture was incubated for 20 min at 46°C and then diluted 10-fold with dialysis buffer (10 mM Tris–HCl, 10 mM MgSO_4_, 25 mM NaCl, pH 7.5) at 4°C. After concentration to the starting volume (6 ml) by centrifugation in a 10,000 molecular weight cut-off (MWCO) device (Amicon Centriprep YM10, Millipore Corporation, Bedford MA, USA), the concentrate was dialyzed against 4 L of dialysis buffer for 24 h in a 10,000 MWCO cassette (Pierce, Rockford, IL, USA). After dialysis, the sample was processed three times on an immobilized DNase 1 F7_M_ matrix column (MoBiTec, Göttingen, Germany) with elution by gravity. The eluate was dialyzed as before, against two 4 L volumes of the same dialysis buffer to remove the cleaved DNA fragments and then concentrated to 0.5 ml by centrifugation in a 10,000 MWCO device (Centriprep YM10) and stored at -20°C. The protein concentration was estimated from its absorbance at 280 nm at 1.59 mg/ml.

Protein samples were suspended in 8 M urea and 100 mM Tris pH 8.5, reduced with 100 mM TCEP for 30 min followed by cysteine alkylation with 55 mM iodoacetamide for another 30 min in the dark. The mixture was then diluted to 4 M urea by adding 100 mM Tris buffer pH 8.5 (and CaCl_2_ was added to ensure tryptic specificity at 2 mM). Trypsin was then used to digest the protein samples at 37°C for 24 hrs (1:100 enzyme:sample). The digestion was stopped with the addition of formic acid to 4% (v/v) prior to column loading.

The protein digest was pressure-loaded onto a column containing 4 cm of 5 μm C18 resin packed into 250 μm inner diameter fused silica capillary with a M-520 0.5 μm filter assembly (IDEX Health & Science LLC, Oak Harbor, WA), followed by desalting with 0.1% formic acid in 5% acetonitrile. The loaded C18 column was then connected to 100 μm (i.d.) analytical column consisting of 4 cm of packed 5 μm strong cation exchange resin (SCX Partisphere, Whatman GE Healthcare) and 10 cm of packed C18 resin (Polymicro Technologies, Phoenix, AZ) with a 5 μm laser pulled tip. The column assembly was placed inline and LC/LC-MS/MS was carried out as described earlier [93], using a 12-step separation with an Agilent HP1100 system connected to a LCQ Deca ion trap mass spectrometer (Thermo Scientific).

Tandem mass spectra were collected in a data-dependent pattern by collecting one full MS scan (m/z range = 400–1400) followed by MS/MS spectra of the three most abundant precursor ions. The MS/MS spectra were then processed and searched against the protein database (NCBI) using the SEQUEST algorithm (http://fields.scripps.edu/sequest/). All subsequent filtering and comparisons of identifications were made using DTASelect and Contrast software [94].

## Abbreviations

AA: Amino acid; BLAST: Basic local alignment search tool; CFU: Colony forming unit, a measure of the number of viable bacterial cells; DNase: Deoxyribonuclease; ECOR: *Escherichia coli* collection of reference; gp: Gene product; LC-MS: Liquid chromatography–mass spectrometry; MAFFT: Multiple alignment using fast fourier transform; MOI: Multiplicity of Infection, ratio of infective phage particles to vulnerable hosts; MS-MS: Tandem mass spectrometry; MudPIT: Multi-dimensional protein identification technology; PDB: Research collaboratory for structural bioinformatics (rcsb) protein data bank; PFU: Plaque forming unit, a measure of the number of viable viral particles; RNase: Ribonuclease; TCEP: (tris(2-carboxyethyl)phosphine); TMHMM: TransMembrane prediction using Hidden Markov Models; TSB: Tryptic soy broth.

## Competing interests

The authors have no competing interests to disclose.

## Authors’ contributions

AMK and RPJ designed and guided the project; TW, RPJ, and AM isolated rV5 and with KF, AM and EJL were involved in the initial characterization of this phage; DMM and HWA performed the electron microscopy; EJL and AM arranged for the sequencing; EJL carried out the PFGE; JM and JY conducted the proteomic analyses; and AMK did the annotation. AMK did most of the writing of the paper and all authors read and approved the final manuscript.

## Supplementary Material

Additional file 1: Table S1 Sensitivity of reference strains of 12 common phage types of *E. coli *O157:H7 to lysis by phage rV5. Click here for file

Additional file 2: Table S2 Sensitivity of ECOR strains to lysis by phage rV5. Click here for file

Additional file 3: Table S3 rV5 genes, their products, homologs and potential function. Click here for file

Additional file 4: Table S4 Predicted promoters and rho-independent terminators found in the rV5 genome. Click here for file

Additional file 5: Table S5 MS data on rV5. Click here for file
