# Supplementary material for: The host-range, genomics and proteomics of Escherichia coli O157:H7 bacteriophage rV5
Source: Virol J. 2013 Mar 6;10:76. doi: 10.1186/1743-422X-10-76 (PMC3606486; doi:10.1186/1743-422X-10-76)
Supplement: Additional file 1: Table S1 — Sensitivity of reference strains of 12 common phage types of E. coli O157:H7 to lysis by phage rV5. [file 1743-422X-10-76-S1.doc]

**Additional file 1, Table S1:** Sensitivity^a^ of reference strains of 12 common phage types of *E. coli* O157:H7 to lysis by phage rV5.

| **Strain No.** | **O157 Phage Type** | **Sensitivity to rV5** |
| --- | --- | --- |
| EC990299 | 1 | 2+^b^ |
| EC990300 | 2 | - |
| EC990295 | 4 | - |
| EC990705 | 8 | 4+ |
| EC990298 | 14 | 3+ |
| EC990293 | 21 | - |
| EC990296 | 23 | - |
| EC990294 | 24 | 4+ |
| EC990301 | 31 | - |
| EC990302 | 32 | - |
| EC990297 | 33 | 1+ |
| EC990730 | 87 | 3+ |
|  |  |  |

^a^Tested by spotting 10^5^ PFU of phage rV5 onto freshly seeded lawns of bacteria on agar plates. After 20 h incubation at 37°C, the proportion (%) of the total area within the spots showing lysis as plaques, or semi-confluent and confluent zones of lysis was recorded. Where present, lytic activity of phage rV5 was in all cases manifest as clear lysis.

^b^1+ = approx. 10-25% lysis; 2+ = approx. 25-50% lysis; 3+ = approx. 50-75% lysis; 4+ = approx. 75-100% lysis; - = no lysis.
