# Supplementary material for: The host-range, genomics and proteomics of Escherichia coli O157:H7 bacteriophage rV5
Source: Virol J. 2013 Mar 6;10:76. doi: 10.1186/1743-422X-10-76 (PMC3606486; doi:10.1186/1743-422X-10-76)
Supplement: Additional file 2: Table S2 — Sensitivity of ECOR strains to lysis by phage rV5. [file 1743-422X-10-76-S2.doc]

**Additional file 2, Table S2:** Sensitivity^a^ of ECOR strains to lysis by phage rV5.

| **Strain No.** | **ECOR No.** | **Serotype** | **Lysis^b^ by phage rV5** |
| --- | --- | --- | --- |
| EC990774 | 1 | O144:H4 | - |
| EC990775 | 2 | O?:H32 | - |
| EC990776 | 3 | O1:H32 | - |
| EC990777 | 4 | OR:H20 | p |
| EC990778 | 5 | O?:H6 | - |
| EC990779 | 6 | O173:H? | 1+ |
| EC990780 | 7 | O8:H45 | - |
| EC990781 | 8 | O86:H2 | - |
| EC990782 | 9 | O167:NM | 1+ |
| EC990783 | 10 | O6:H10 | - |
| EC990784 | 11 | O10:NM | - |
| EC990785 | 12 | O?:H32 | - |
| EC990786 | 13 | OR:H25 | 3+ |
| EC990787 | 14 | O71:H4 | p |
| EC990788 | 15 | O25:H30 | 1+ |
| EC990789 | 16 | O9:H10 | 1+ |
| EC990790 | 17 | O29:NM | - |
| EC990791 | 18 | O?:H? | - |
| EC990792 | 19 | O89:H? | - |
| EC990793 | 20 | O121:H? | - |
| EC990794 | 21 | O121:H11 | - |
| EC990795 | 22 | O150:H28 | - |
| EC990796 | 23 | O25:H1 | - |
| EC990797 | 24 | O15:NM | - |
| EC990798 | 25 | O127:H40 | - |
| EC990799 | 26 | O104:H21 | - |
| EC990800 | 27 | O104:H21 | - |
| EC990801 | 28 | O014:H2 | p |
| EC990802 | 29 | O150:H21 | 1+ |
| EC990803 | 30 | O113:H21 | 1+ |
| EC990804 | 31 | O79:H25 | - |
| EC990805 | 32 | O25:H1 | - |
| EC990806 | 33 | O7:H21 | 1+ |
| EC990807 | 34 | O88:NM | - |
| EC990808 | 35 | O1:NM | - |
| EC990809 | 36 | O1:NM | - |
| EC990810 | 37 | O55:H7 | - |
| EC990811 | 38 | O7:NM | - |
| EC990812 | 39 | O7:NM | - |
| EC990813 | 40 | O7:NM | - |
| EC990814 | 41 | O7:NM | - |
| EC990815 | 42 | O87:H26 | 1+ |
| EC990816 | 43 | O?:H18 | p |
| EC990817 | 44 | O17:H34 | - |
| EC990818 | 45 | O?H2 | - |
| EC990819 | 46 | O1:NM | - |
| EC990820 | 47 | O17:H18 | 1+ |
| EC990821 | 48 | O23:H15 | - |
| EC990822 | 49 | O2:H4 | - |
| EC990823 | 50 | O2:H4 | p |
| EC990824 | 51 | O25:H1 | - |
| EC990825 | 52 | O25:H1 | - |
| EC990826 | 53 | O4:H5 | - |
| EC990827 | 54 | O25:H1 | - |
| EC990828 | 55 | O25:H1 | - |
| EC990829 | 56 | O6:H1 | - |
| EC990830 | 57 | O2:H1 | - |
| EC990831 | 58 | O112:H8 | - |
| EC990832 | 59 | O2:H4 | - |
| EC990833 | 60 | O4:H5 | - |
| EC990834 | 61 | O2:H4 | - |
| EC990835 | 62 | O2:H4 | - |
| EC990836 | 63 | OR:NM | - |
| EC990837 | 64 | O75:NM | - |
| EC990838 | 65 | O8:H10 | - |
| EC990839 | 66 | O4:H40 | - |
| EC990840 | 67 | O141:H49 | 2+ |
| EC990841 | 68 | O25:H21 | - |
| EC990842 | 69 | O86:H10 | - |
| EC990843 | 70 | O78:NM | - |
| EC990844 | 71 | O8:H30 | - |
| EC990845 | 72 | O8:H30 | - |

^a^Tested by spotting 10^5^ PFU of rV5 onto freshly seeded lawns of bacteria on appropriate agar plates. After 20 h incubation at 37°C, the proportion (%) of the total area within the spots showing plaques or semi-confluent and confluent zones of clear or opaque lysis was recorded.

^b^p: = 1-approx. 100 plaques; 1+ = approx. 10-25% lysis; 2+ = approx. 25-50% lysis; 3+ = approx. 50-75% lysis; 4+ = approx. 75-100% lysis; - = no lysis.
