# Supplementary material for: The host-range, genomics and proteomics of Escherichia coli O157:H7 bacteriophage rV5
Source: Virol J. 2013 Mar 6;10:76. doi: 10.1186/1743-422X-10-76 (PMC3606486; doi:10.1186/1743-422X-10-76)
Supplement: Additional file 3: Table S3 — rV5 genes, their products, homologs and potential function. [file 1743-422X-10-76-S3.doc]

**Additional file 3, Table S3:** rV5 genes, their products, homologs and potential function.

| **Gene** | **Product** | **From** | **To** | **Protein Size(aa) / Molecular Mass(kDa) / pI** | **Homologs & Pfam motifs** | **E value** |
| --- | --- | --- | --- | --- | --- | --- |
| *1* | rIIA - rIIA protector from prophage-induced early lysis | 1 | 1983< | 660 / 75811 / 8.2 | YP_002456024.1| RIIA [Erwinia phage phiEa21-4]; PF13589.1 HATPase_c_3 | 9e-31 |
| *2* | cell wall hydrolase | 1980 | 2480< | 166 / 19052 / 10.0 | PF07486.7 Hydrolase_2 |  |
| *3* | CHP | 2530 | 2910< | 126 / 14634 / 4.3 | YP_006911.1| hypothetical protein T5.083 [Enterobacteria phage T5] | 6e-22 |
| *4* | MoxR - ATPase | 2920 | 4104< | 394 / 43388 / 4.9 | YP_004060403.1| AAA ATPase central domain-containing protein [Sulfuricurvum  kujiense DSM 16994] | 3e-38 |
| *5* |  | 4104 | 4373< | 89 / 10822 / 5.3 | - |  |
| *6* |  | 4418 | 4798< | 126 / 14839 / 4.2 | - |  |
| *7* |  | 4801 | 5058< | 85 / 9775 / 4.1 | - |  |
| *8* |  | 5058 | 5414< | 118 / 13253 / 4.0 | - |  |
| *9* |  | 5428 | 5700< | 90 / 10154 / 6.6 | - |  |
| *10* |  | 5703 | 6602< | 299 / 33689 / 4.9 | - |  |
| *11* |  | 6787 | 7722 | 311 / 35958 / 4.6 | - |  |
| *12* | CHP | 7756 | 9081 | 441 / 49521 / 8.5 | YP_002514352.1| hypothetical protein Tgr7_2285 [Thioalkalivibrio sulfidophilus  HL-EbGr7]; PF13203.1 DUF2201_N | 3e-17 |
| *13* |  | 8972 | 9217 | 81 / 9522 / 5.7 | - |  |
| *14* |  | 9233 | 9490 | 85 / 9397 / 4.5 | - |  |
| *15* |  | 9490 | 9753 | 87 / 8749 / 4.4 | - |  |
| *16* |  | 9755 | 9988 | 77 / 8749 / 4.4 | - |  |
| *17* |  | 10005 | 10454 | 149 / 17422 / 5.1 | - |  |
| *18* |  | 10451 | 10696 | 81 / 9298 / 8.2 | - |  |
| *19* |  | 10699 | 11265 | 188 / 21359 / 5.6 | - |  |
| *20* |  | 11323 | 11598 | 91 / 10429 / 4.7 | - |  |
| *21* | CHP | 11601 | 12065 | 154 / 17966 / 4.4 | YP_001671792.1| hypothetical protein phi32_47 [Enterobacteria phage phiEco32] | 1e-10 |
| *22* |  | 12071 | 12241 | 56 / 6888 / 8.6 | - |  |
| *23* |  | 12114 | 12263 | 49 / 5495 / 9.4 | - |  |
| *24* |  | 12292 | 12672 | 126 / 14226 / 9.3 | - |  |
| *25* | CHP | 12836 | 13243 | 135 / 15492 / 5.2 | YP_398953.1| hypothetical protein rtp9 [Enterobacteria phage RTP] | 1e-04 |
| *26* | CHP | 13247 | 13447 | 66 / 7543 / 4.2 | YP_002922792.1| hypothetical protein WV8_gp011 [Enterobacteria phage WV8] | 2e-28 |
| *27* | CHP | 13497 | 19133< | 1878 / 208334 / 6.5 | NP_669509.1| hypothetical protein y2199 [Yersinia pestis KIM 10]; PF02368.13  Big_2; smart00635 BID_2 | 1e-04 |
| *28* |  | 19182 | 20927< | 581 / 62673 / 4.7 | - |  |
| *29* |  | 20937 | 21203< | 88 / 10012.6 / 7.3 | - |  |
| *30* | tail fibre protein | 21215 | 22258< | 347 / 38393 / 5.7 | CAZ39578.1| hypothetical protein [Erwinia phage phiAT1] | 1e-06 |
| *31* | membrane protein | 22348 | 22662< | 104 / 11948 / 7.3 | 1 TMD |  |
| *32* | tail fibre assembly protein | 22674 | 23258< | 194 / 22538 / 4.5 | ADX32387.1| tail fiber assembly protein [Cronobacter phage ESSI-2]; PF02413.12  Caudo_TAP | 2e-10 |
| *33* | tail fiber protein | 23273 | 24313< | 346 / 38292 / 5.5 | CAZ39578.1| hypothetical protein [Erwinia phage phiAT1] | 2e-15 |
| *34* | structural protein (MS evidence) | 24326 | 26398< | 690 / 79100 / 5.0 | ADX03900.1| Putative uncharacterized protein [Acinetobacter baumannii 1656-2]; PF03160.9   Calx-beta; smart00237 Calx_beta | 4e-96 |
| *35* | CHP | 26398 | 27084< | 228 / 25397 / 4.4 | ADX88569.1| hypothetical protein TUST1-159_00345 [Vibrio phage ICP1_2006_B] | 2e-04 |
| *36* | baseplate assembly protein | 27096 | 28586< | 496 / 54737 / 4.8 | YP_002922862.1| hypothetical protein WV8_gp080 [Enterobacteria phage WV8]; PF04865.9   Baseplate_J | 2e-24 |
| *37* | tail fibre protein (host-specificity) | 28692 | 32531< | 1279 / 140069 / 5.3 | YP_002150697.1| phage tail protein [Proteus mirabilis HI4320]; PF13550.1   Phage-tail_3; PF12421.3  DUF3672; TIGR02169 SMC_prok_A | e-164 |
| *38* | CHP | 32531 | 32956< | 141 / 16653 / 5.3 | NP_892065.1| hypothetical protein PY54p19 [Yersinia phage PY54] | 1e-21 |
| *39* | CHP | 32956 | 33507< | 183 / 19935 / 4.6 | ZP_03804004.1| hypothetical protein PROPEN_02380 [Proteus penneri ATCC 35198] | 3e-30 |
| *40* | CHP | 33509 | 34099< | 196 / 21987 / 9.2 | NP_892062.1| hypothetical protein PY54p16 [Yersinia phage PY54] | 9e-21 |
| *41* | structural protein (MS evidence); L-shaped tail fiber protein | 34199 | 38017< | 1272 / 139487 / 5.6 | AEE56368.1| L-shaped tail fiber protein [Escherichia coli UMNK88]; PF13884.1   Peptidase_S74 | 1e-44 |
| *42* | tail associated protein | 38053 | 40650< | 865 / 97739 / 5.7 | YP_001438401.1| hypothetical protein ESA_02316 [Cronobacter sakazakii ATCC BAA-894] | e-127 |
| *43* | tail fiber protein | 40652 | 41320< | 222 / 24176 / 4.2 | YP_001438402.1| hypothetical protein ESA_02317 [Cronobacter sakazakii ATCC BAA-894] | 4e-17 |
| *44* | CHP | 41331 | 42046< | 238 / 27544 / 4.4 | ADF58207.1| hypothetical protein PJG4_081 [Pseudomonas phage JG004] | 2e-07 |
| *45* | baseplate assembly protein | 42059 | 42736< | 225 / 24524/ 4.7 | YP_249015.1| hypothetical protein NTHI1548 [Haemophilus influenzae 86-028NP] | 9e-10 |
| *46* | CHP | 42736 | 43740< | 334 / 36979 / 8.8 | CAZ39575.1| hypothetical protein [Erwinia phage phiAT1] | 2e-84 |
| *47* |  | 43740 | 44120< | 126 / 14866 / 4.4 | - |  |
| *48* | CHP | 44133 | 45017< | 294 / 32763 / 5.4 | YP_851468.1| hypothetical protein APECO1_1716 [Escherichia coli APEC O1] | 4e-16 |
| *49* | minor tail protein | 45123 | 47459< | 778 / 85633 / 9.8 | YP_001462506.1| hypothetical protein EcE24377A_1404 [Escherichia coli E24377A] | 2e-19 |
| *50* |  | 47514 | 47882< | 122 / 13923 / 8.5 | - |  |
| *51* |  | 47774 | 48247< | 157 / 17823 / 4.3 | - |  |
| *52* | structural protein (MS evidence); tail tube protein | 48410 | 48883< | 157 / 17183 / 4.5 | ADE87495.1| phage structure protein [Deep-sea thermophilic phage D6E]; PF11681.3   DUF3277 | 3e-12 |
| *53* | structural protein (MS evidence); tail sheath protein | 48894 | 50270< | 458 / 50271 / 5.0 | YP_004327204.1| hypothetical protein PsPhPAKP1_gp059 [Pseudomonas phage PAK_P1]; PF11863.3   DUF3383 | 2e-38 |
| *54* |  | 50343 | 50894< | 183 / 20404 / 5.0 | - |  |
| *55* |  | 50894 | 51319< | 141 / 15980 / 5.0 | - |  |
| *56* | CHP | 51335 | 51793< | 152 / 17269 / 9.0 | YP_004327201.1| hypothetical protein PsPhPAKP1_gp056 [Pseudomonas phage PAK_P1] | 5e-09 |
| *57* |  | 51847 | 52467< | 206 / 23474 / 4.4 | - |  |
| *58* |  | 52527 | 53108< | 193 / 20919 / 4.1 | - |  |
| *59* |  | 53131 | 53355< | 74 / 8630 / 7.1 | - |  |
| *60* | major head protein (MS evidence) | 53437 | 54447< | 336 / 37873 / 5.9 | ADX88168.1| putative major head protein [Vibrio phage ICP1_2006_D]; PF03864.10  Phage_cap_E | 4e-48 |
| *61* | structural protein (MS evidence); head decoration protein | 54496 | 54885< | 129 / 13758 / 5.8 | - |  |
| *62* | structural protein (MS evidence) | 54906 | 55883< | 325 / 3638 / 4.6 | ADX88170.1| hypothetical protein TUST1-191_00620 [Vibrio phage ICP1_2006_D] | 2e-11 |
| *63* | CHP | 55883 | 56455< | 190 / 21663 / 4.8 | CAZ39591.1| hypothetical protein [Erwinia phage phiAT1] | 3e-21 |
| *64* | portal protein | 56370 | 57926< | 518 / 57129 / 6.3 | CAZ39587.1| hypothetical protein [Erwinia phage phiAT1] | 3e-83 |
| *65* | HNH homing endonuclease | 58995 | 59690< | 231 / 26544 / 8.5 | CAZ39573.1| hypothetical protein [Erwinia phage phiAT1]; PF13384.1 HTH_23; PF13392.1  HNH_3 | 2e-34 |
| *64abc* | TerL; terminase, large subunit (spliced) |  |  | 515 / 58613 / 5.8 | YP_004306750.1| putative terminase large subunit [Pseudomonas phage KPP10]; PF03237.10  Terminase_6 | 1e-93 |
| *66* |  | 60872 | 61159< | 95 / 10412 / 9.0 | - |  |
| *67* | membrane protein | 61156 | 61554< | 132 / 15087 / 6.0 | 1 TMD |  |
| *68* |  | 61660 | 61929< | 89 / 10370 / 4.7 | - |  |
| *69* | tRNA^Arg^ | 61976 | 62052< |  |  |  |
| *70* | tRNA^Tyr^ | 62064 | 62138< |  |  |  |
| *71* | tRNA^Thr^ | 62140 | 62215< |  |  |  |
| *72* | tRNA^Met^ | 62623 | 62709< |  |  |  |
| *73* | tRNA^Pro^ | 62714 | 62788< |  |  |  |
| *74* |  | 62885 | 63223< | 112 / 13099 / 4.9 | - |  |
| *75* |  | 63568 | 63810< | 80 / 9253 / 4.0 | - |  |
| *76* |  | 63887 | 64075< | 62 / 7194 / 4.9 | - |  |
| *77* |  | 64086 | 64664< | 192 / 22497 / 6.9 | - |  |
| *78* |  | 64759 | 64929< | 56 / 6525 / 12.0 | - |  |
| *79* | structural protein (MS evidence) | 65308 | 65658< | 116 / 12802 / 9.4 | - |  |
| *80* | phosphoesterase | 65706 | 66260< | 184 / 21562 / 6.8 | ZP_03610184.1| metallophosphoesterase [Campylobacter rectus RM3267]; PF12850.2 Metallophos_2 | 5e-28 |
| *81* | ATPase | 66268 | 66678 | 136 / 15757 / 6.9 | YP_001964067.1| hypothetical protein LBF_3019 [Leptospira biflexa serovar Patoc strain 'Patoc 1 (Ames)']; PF13671.1   AAA_33 | 4e-27 |
| *82* | RNA ligase | 66779 | 67714 | 311 / 36089 / 6.3 | YP_004327167.1| hypothetical protein PsPhPAKP1_gp022 [Pseudomonas phage PAK_P1]; PF09511.5 RNA_lig_T4_1 | 9e-54 |
| *83* |  | 67711 | 68061 | 116 / 13712 / 5.0 | - |  |
| *84* | NAD-dependent protein deacetylases SIR2 family; SP | 68070 | 68831 | 253 / 28555 / 4.8 | YP_006930.1| putative Sir2-like protein [Enterobacteria phage T5]; PF02146.12 SIR2 | e-102 |
| *85* |  | 68845 | 69558 | 237 / 27395/ 6.0 | - |  |
| *86* |  | 69622 | 69930 | 102 / 12024 / 6.3 | - |  |
| *87* |  | 69917 | 70186 | 89 / 10363 / 4.5 | - |  |
| *88* | DNA ligase | 70186 | 71451 | 421 / 48590 / 5.7 | AEK81958.1| ATP-dependent DNA ligase [Salmonella phage 7-11]; PF01068.16  DNA_ligase_A_M | 3e-69 |
| *89* | lysin (lysozyme) | 71575 | 72045 | 156 / 17421 / 9.3 | YP_001742044.1| lysozyme [Salmonella phage E1]; PF00959.14 Phage_lysozyme | 5e-71 |
| *90* |  | 72109 | 72315 | 68 / 7603 / 4.8 |  |  |
| *91* | HNH endonuclease | 72325 | 72789 | 154 / 17804 / 8.2 | YP_001520719.1| hypothetical protein AM1_A0059 [Acaryochloris marina MBIC11017] | 8e-40 |
| *92* |  | 72782 | 73072 | 96 / 11210 / 9.8 | - |  |
| *93* |  | 73132 | 73440 | 102 / 11879 / 6.2 | - |  |
| *94* | 5'-3' exonuclease | 73433 | 74572 | 379 / 43307 / 8.2 | YP_004306803.1| putative exodeoxyribonuclease [Pseudomonas phage KPP10] | 3e-37 |
| *95* |  | 74572 | 74904 | 110 / 12580 / 3.9 | - |  |
| *96* |  | 74901 | 75530 | 209 / 23287 / 8.1 | - |  |
| *97* | EndoVII packaging and recombination endonuclease | 75509 | 76087 | 192 / 22636 / 6.8 | NP_899379.1| EndoVII packaging and recombination endonuclease [Vibrio phage KVP40]; PF02945.10 Endonuclease_7 | 1e-09 |
| *98* | CHP | 76026 | 76247 | 73 / 8825 / 7.2 | YP_004347282.1| hypothetical protein LAU_0319 [Lausannevirus] | 5e-18 |
| *99* | CHP | 76260 | 77243 | 327 / 38334 / 6.2 | YP_004306808.1| putative DNA polymerase [Pseudomonas phage KPP10] | 4e-51 |
| *100* | adenine methyltransferase | 77289 | 78107 | 272 / 30996 / 6.1 | YP_615201.1| hypothetical protein Sala_0144 [Sphingopyxis alaskensis RB2256]; pfam13659 Methyltransf_26 | 9e-41 |
| *101* |  | 78070 | 78528 | 152 / 17150 / 10.0 | - |  |
| *102* |  | 78556 | 78747 | 63 / 7149 / 4.0 | - |  |
| *103* | CHP | 78747 | 79361 | 204 / 22792 / 5.1 | YP_003883.1| hypothetical protein T1p62 [Enterobacteria phage T1] | 2e-22 |
| *104* | 5’,3’-deoxy-ribonucleotidase | 79355 | 79948 | 197 / 22891 / 5.6 | YP_004251113.1| hypothetical protein [Vibrio phage ICP1]; PF06941.7 NT5C | 4e-09 |
| *105* |  | 79950 | 80150 | 66 / 7549 / 9.2 | - |  |
| *106* | thymidylate synthase | 80150 | 81133 | 327 / 37635 / 6.4 | ADX88687.1| putative thymidylate synthase [Vibrio phage ICP1_2006_B]; PF02511.10 Thy1 | e-109 |
| *107* |  | 81232 | 81693 | 153 / 17535 / 8.1 | - |  |
| *108* |  | 81704 | 81934 | 76 / 8985 / 4.0 | - |  |
| *109* | NrdA, rNDP reductase, alpha subunit | 81931 | 84246 | 771 / 87359 / 6.2 | YP_002456126.1| putative ribonucleoside triphosphate reductase alpha chain [Erwinia phage phiEa21-4]; PF00317.16  Ribonuc_red_lgN;PF02867.10 Ribonuc_red_lgC | 0 |
| *110* | NrdB, rNDP reductase, beta subunit | 84286 | 85374 | 362 / 41786 / 4.4 | AEJ81333.1| ribonucleoside triphosphate reductase, beta chain [Erwinia phage  vB_EamM-M7]; PF00268.16 Ribonuc_red_sm | e-134 |
| *111* | glutaredoxin | 85378 | 85656 | 92 / 10355 / 7.1 | YP_003913559.1| glutaredoxin, GrxA family [Ferrimonas balearica DSM 9799]; PF00462.19 Glutaredoxin | 5e-11 |
| *112* | NrdD, anaerobic rNTP reductase, large subunit | 85653 | 87776 | 707 / 79055 / 5.8 | YP_003580093.1| NrdD anaerobic ribonucleotide reductase subunit [Klebsiella phage KP15]; PF13597.1 NRDD | 0 |
| *114* | membrane protein | 87773 | 88000 | 75 / 8380 / 10.0 | 1 TMD |  |
| *115* |  | 87970 | 88074 | 34 / 4171 / 11.0 | - |  |
| *116* |  | 88106 | 88342 | 78 / 9106 / 4.4 | - |  |
| *117* | NrdG, anaerobic rNTP reductase, small subunit | 88339 | 88812 | 157 / 17436 / 5.6 | YP_002854412.1| anaerobic nucleotide reductase subunit [Enterobacteria phage RB14]; PF13353.1 Fer4_12; PF04055.16 Radical_SAM | 4e-43 |
| *118* |  | 88875 | 88988 | 37 / 4482 / 9.8 | - |  |
| *119* |  | 88985 | 89335 | 116 / 13271 / 5.1 | - |  |
| *120* | PhoH-like protein | 89374 | 90162 | 262 / 29559 / 9.1 | AEK81995.1| PhoH-like protein [Salmonella phage 7-11]; PF02562.11 PhoH | 2e-41 |
| *121* |  | 90266 | 90577 | 103 / 11973 / 8.6 | - |  |
| *122* | ClpP (ATP-dependent) protease subunit | 90638 | 91687 | 349 / 38541 / 5.0 | ADX89162.1| ClpP ATP-dependent protease subunit [Vibrio phage ICP1_2005_A]; PF00574.18 CLP_protease | 3e-20 |
| *123* | CHP | 91739 | 92272 | 177 / 20533 / 4.6 | YP_001445151.1| hypothetical protein VIBHAR_01959 [Vibrio harveyi ATCC BAA-1116] | 3e-29 |
| *124* |  | 92269 | 92553 | 94 / 10222 / 6.6 | - |  |
| *125* |  | 92589 | 92828 | 79 / 9520 / 6.3 | - |  |
| *126* |  | 92825 | 93223 | 132 / 14464 / 5.1 | - |  |
| *127* |  | 93263 | 93496 | 77 / 8333 / 5.2 | - |  |
| *128* |  | 93502 | 94026 | 174 / 20913 / 5.8 | - |  |
| *129* | putative holin | 94058 | 94294 | 78 / 8896 / 9.3 | 2 TMD |  |
| *130* |  | 94278 | 94430 | 50 / 5739 / 8.2 | - |  |
| *131* | membrane protein | 94408 | 94527 | 39 / 4265 / 6.4 | 1 TMD |  |
| *132* |  | 94514 | 94699 | 61 / 7464 / 4.1 | - |  |
| *133* | structural protein (MS evidence) | 94702 | 95157 | 151 / 17626 / 8.7 | 1 TMD |  |
| *134* | membrane protein | 95218 | 95565 | 115 / 13239 / 4.7 | 1 TMD |  |
| *135* |  | 95562 | 95849 | 95 / 10840 / 9.0 | - |  |
| *136* |  | 95846 | 96046 | 66 / 7819 / 4.5 | - |  |
| *137* | structural protein (MS evidence) | 96066 | 96251 | 61 / 7231 / 4.8 | - |  |
| *138* |  | 96347 | 96643 | 98 / 11221 / 9.0 | - |  |
| *139* |  | 96646 | 96927 | 93 / 10771 / 8.9 | - |  |
| *140* |  | 96927 | 97151 | 74 / 8149 / 3.9 | - |  |
| *141* |  | 97141 | 97395 | 84 / 9301 / 4.5 | - |  |
| *142* |  | 97408 | 97806 | 132 / 15107 / 8.1 | - |  |
| *143* |  | 97824 | 98114 | 96 / 10647 / 4.1 | - |  |
| *144* | sigma 54 modulation factor | 98197 | 98514 | 105 / 11501 / 8.6 | YP_002236385.1| sigma(54) modulation protein [Klebsiella pneumoniae 342]; PF02482.14 Ribosomal_S30AE | 2e-11 |
| *145* | membrane protein | 98524 | 98625 | 33 / 3614 / 6.3 | 1 TMD |  |
| *146* |  | 98652 | 98921 | 89 / 9892 / 4.3 | - |  |
| *147* |  | 99013 | 99276 | 87 / 9632 / 3.9 | - |  |
| *148* |  | 99281 | 99613 | 110 / 12128 / 4.5 | - |  |
| *149* |  | 99613 | 100008 | 131 / 14961 / 4.6 | - |  |
| *150* |  | 100088 | 100345 | 85 / 9480 / 4.0 | - |  |
| *151* |  | 100346 | 100696 | 116 / 12771 / 3.6 | - |  |
| *152* | structural protein (MS evidence) | 100696 | 101025 | 109 / 12340 / 4.4 | - |  |
| *153* |  | 101025 | 101237 | 70 / 7639 / 5.9 | - |  |
| *154* |  | 101161 | 101295 | 44 / 5355 / 9.6 | - |  |
| *155* |  | 101404 | 101667 | 87 / 10064 / 8.6 | - |  |
| *156* | membrane protein | 101696 | 101836 | 46 / 5071 / 4.4 | 1 TMD |  |
| *157* |  | 101823 | 102074 | 83 / 9347 / 4.1 | - |  |
| *158* |  | 102103 | 102408 | 101 / 11906 / 5.2 | - |  |
| *159* |  | 102464 | 102796 | 110 / 12818 / 5.0 | - |  |
| *160* |  | 102805 | 103104 | 99 / 11523 / 5.5 | - |  |
| *161* |  | 103137 | 103295 | 52 / 6267 / 9.1 | - |  |
| *162* |  | 103372 | 103677 | 101 / 11474 / 4.2 | - |  |
| *163* |  | 103732 | 104013 | 93 / 10454 / 4.0 | - |  |
| *164* |  | 106618 | 107037< | 139 / 16069 / 6.1 | - |  |
| *165* |  | 107184 | 107447< | 87 / 10692 / 8.6 | - |  |
| *166* |  | 107525 | 107743< | 72 / 7943 / 8.9 | - |  |
| *167* |  | 107804 | 108076< | 90 / 10548 / 4.4 | - |  |
| *168* |  | 107950 | 108111< | 53 / 6103 / 10.3 | - |  |
| *169* |  | 108289 | 108480< | 63 / 7449 / 5.2 | - |  |
| *170* | membrane protein | 108489 | 108623< | 44 / 4917 / 9.6 | 1 TMD |  |
| *171* |  | 108626 | 108910< | 94 / 10347 / 5.2 | - |  |
| *172* |  | 109002 | 109217< | 71 / 8149 / 9.5 | - |  |
| *173* | membrane protein | 109227 | 109367< | 46 / 5022 / 5.7 | 1 TMD |  |
| *174* |  | 109422 | 109601< | 59 / 6952 / 4.3 | - |  |
| *175* |  | 109678 | 109911< | 77 / 8718 / 8.4 | - |  |
| *176* |  | 109966 | 110250< | 94 / 10803 / 4.0 | - |  |
| *177* |  | 110475 | 110681< | 68 / 7882 / 4.7 | - |  |
| *178* |  | 110678 | 110776< | 32 / 3841 / 5.5 | - |  |
| *179* |  | 110868 | 111068< | 66 / 7520 / 7.3 | - |  |
| *180* |  | 111043 | 111168< | 41 / 4903 / 10.2 | - |  |
| *181* | membrane protein | 111210 | 111329< | 39 / 4454 / 8.2 | 1 TMD |  |
| *182* |  | 111326 | 111571< | 81 / 9543 / 8.7 | - |  |
| *183* | membrane protein | 111549 | 111920< | 123 / 13952 / 8.5 | 1-2 TMD |  |
| *184* |  | 111985 | 112365< | 126 / 14569 / 7.2 | - |  |
| *185* | CHP | 112455 | 113078< | 207 / 24094 / 4.3 | ZP_00964682.1| hypothetical protein NAS141_01396 [Sulfitobacter sp. NAS-14.1] | 2e-05 |
| *186* |  | 113153 | 113413< | 86 / 10234 / 9.9 | - |  |
| *187* |  | 113509 | 113625< | 38 / 4062 / 11.2 | - |  |
| *188* |  | 113607 | 113732< | 41 / 4874 / 11.7 | - |  |
| *189* |  | 113659 | 113856< | 65 / 8220 / 10.6 | PF08200.6  Phage_1_1 |  |
| *190* |  | 113846 | 114199< | 117 / 13354 / 8.1 | - |  |
| *191* |  | 114276 | 114521< | 81 / 9246 / 8.7 | - |  |
| *192* |  | 114611 | 114961< | 116 / 13639 / 3.7 | - |  |
| *193* | membrane protein | 115042 | 115161< | 39 / 4425 / 8.2 | 1 TMD |  |
| *194* |  | 115242 | 115646< | 134 / 15467 / 9.4 | - |  |
| *195* |  | 115729 | 116127< | 132 / 14888 / 5.8 | - |  |
| *196* |  | 116195 | 116476< | 94 / 10960/ 10.0 | - |  |
| *197* |  | 116486 | 116611< | 41 / 5082 / 10.0 | - |  |
| *198* |  | 116782 | 117060< | 92 / 10186 / 8.5 | - |  |
| *199* |  | 117409 | 117621< | 70 / 8145 / 10.2 | - |  |
| *200* | membrane protein | 117667 | 117996< | 109 / 12697 / 9.4 | 1 TMD |  |
| *201* |  | 117993 | 118103< | 36 / 4095 / 8.6 | - |  |
| *202* |  | 118100 | 118192< | 30 / 3583 / 4.4 | - |  |
| *203* |  | 118185 | 118430< | 81 / 8872 / 4.3 | - |  |
| *204* |  | 118451 | 118783< | 110 / 12470 / 4.7 | - |  |
| *205* | CHP | 118791 | 118982< | 63 / 7618 / 6.3 | NP_891831.1| hypothetical protein RB49p260 [Enterobacteria phage RB49] | 1e-37 |
| *206* | CHP | 119046 | 119240< | 64 / 7658 / 8.8 | NP_891661.1| hypothetical protein RB49p090 [Enterobacteria phage RB49] | 2e-33 |
| *207* |  | 119237 | 119449< | 70 / 7919 / 4.7 | - |  |
| *208* |  | 119446 | 119616< | 56 / 6184 / 4.7 | - |  |
| *209* | Tk.4 CHP | 119671 | 120129< | 152 / 16965 / 6.2 | YP_002922165.1| Tk.4 conserved hypothetical protein [Enterobacteria phage JSE]; PF01661.16 Macro | 7e-74 |
| *210* | CHP | 120119 | 120544< | 141 / 16928 / 4.1 | YP_001469407.1| hypothetical protein phi1p064 [Enterobacteria phage Phi1] | 6e-20 |
| *211* |  | 120613 | 120810< | 65 / 7748 / 4.1 | - |  |
| *212* | CHP | 120907 | 121323< | 138 / 15950 / 4.2 | ZP_08379015.1| hypothetical protein ECPG_01013 [Escherichia coli H591] | 6e-69 |
| *213* | HNH homing endonuclease | 121386 | 122009< | 207 / 24488 / 8.9 | AEL79623.1| putative HNH homing endonuclease [Escherichia phage vB_EcoP_G7C] | 3e-23 |
| *214* |  | 121981 | 123252< | 423 / 45726 / 5.6 | - |  |
| *215* |  | 123252 | 123617< | 121 / 13703 / 4.3 | - |  |
| *216* |  | 123701 | 123946< | 81 / 8596 / 3.9 | - |  |
| *217* | CHP | 123964 | 124266< | 100 / 11590 / 4.1 | NP_569476.1| hypothetical protein HCM2.0004c [Salmonella enterica subsp.  enterica serovar Typhi str. CT18] | 1e-04 |
| *218* |  | 124256 | 124645< | 129 / 13791 / 3.9 | - |  |
| *219* |  | 124645 | 124938< | 97 / 11006 / 4.3 | - |  |
| *220* |  | 124969 | 125223< | 84 / 9250 / 4.1 | - |  |
| *221* |  | 125300 | 125671< | 123 / 14625 / 4.7 | - |  |
| *222* |  | 125682 | 125876< | 64 / 7354 / 4.9 | - |  |
| *223* |  | 125873 | 126100< | 75 / 8782/ 4.8 | - |  |
| *224* |  | 126097 | 126459< | 120 / 13729 / 6.3 | - |  |
| *225* |  | 126431 | 126766< | 111 / 12747 / 4.8 | - |  |
| *226* |  | 126577 | 126837< | 86 / 10267 / 10.7 | - |  |
| *227* |  | 126872 | 127030< | 52 / 5998 / 9.7 | - |  |
| *228* | DNA polymerase | 127055 | 129382< | 775 / 89190 / 8.6 | ADX89239.1| DNA polymerase [Vibrio phage ICP1_2001_A]; PF00476.15 DNA_pol_A | 0 |
| *229* |  | 129397 | 129477< | 26 / 2877 / 8.6 | - |  |
| *230* | primase/helicase | 129544 | 131622< | 692 / 77353 / 5.7 | YP_004250998.1| putative primase/helicase [Vibrio phage ICP1]; PF13481.1  AAA_25 | e-173 |
| *231* | Dcm methylase | 131612 | 132376< | 254 / 28575 / 7.1 | AEE56305.1| conserved hypothetical protein [Escherichia coli UMNK88] | 5e-94 |
| *232* |  | 132428 | 132910< | 160 / 18593 / 7.2 | - |  |
| *233* |  | 132923 | 133318< | 131 / 14604 / 4.3 | - |  |
| *234* |  | 133318 | 133623< | 101 / 11718 / 4.3 | - |  |
| *235* | methyltransferase (adenine specific) | 133637 | 134962< | 441 / 49966 / 6.6 | YP_158380.1| adenine specific DNA methylase MOD [Aromatoleum aromaticum EbN1]; PF01555.13 N6_N4_Mtase | e-169 |
| *236* |  | 135024 | 135365< | 113 / 12747 / 4.0 | - |  |
| *237* | ATPase | 135362 | 136732< | 456 / 51379 / 7.6 | YP_001155944.1| hypothetical protein Pnuc_1164 [Polynucleobacter necessarius subsp. asymbioticus QLW-P1DMWA-1]; PF05970.9  PIF1 | 2e-71 |
| *238* | rIIB protector from prophage- induced early lysis | 136732 | 137937< | 401 / 45763 / 4.8 | AEK12529.1| rIIB protector from prophage-induced early lysis [Enterobacteria phage ime09] | 5e-32 |
| CHP = conserved hypothetical protein; HP = hypothetical protein; TMD = transmembrane domain; > ORF transcribed/translated left to right; < ORF transcribed/translated right to left. BLASTP searches conducted 29/08/2011. CD conducted 11/12/2012; only hits with an E-value of <0.0001 were recorded. MS evidence = identified by mass spectroscopy | | | | | | |
