# Supplementary material for: The host-range, genomics and proteomics of Escherichia coli O157:H7 bacteriophage rV5
Source: Virol J. 2013 Mar 6;10:76. doi: 10.1186/1743-422X-10-76 (PMC3606486; doi:10.1186/1743-422X-10-76)
Supplement: Additional file 4: Table S4 — Predicted promoters and rho-independent terminators found in the rV5 genome. [file 1743-422X-10-76-S4.doc]

**Additional file 4, Table S4**

1. Subset of putative promoters discovered using MEME which

maintained 10/12 consensus nucleotides to TTGACA(N15-17)TATAAT


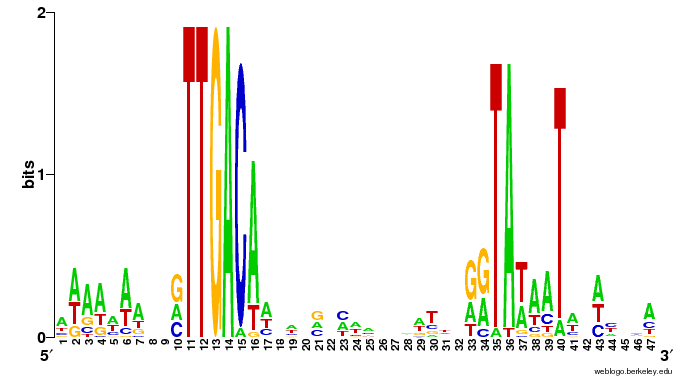


| **Gene** | **Position** | **Strand** | **Sequence** |
| --- | --- | --- | --- |
| P010 | 6636..6681 | - | CAAAAACGTG**TTGAC**TTTACTCTAAATCTCCGA**TATA**G**T**GCACCCA |
| P025 | 12767..12812 | + | AAAATTAAGC**TTGACA**CCAACAATAGATTTGTA**TA**GG**AT**TATCTCA |
| P036 | 28613..28658 | - | TATGCTCAGA**TTGAC**TATTGTTACAAAATT**TG**G**TA**A**AAT**ACCTTGT |
| P051 | 48284..48329 | - | AAAGGTTAAA**TTGACA**ATGCACCATAATCA**TG**G**TA**A**AAT**ACACAAA |
| P074 | 63245..63290 | - | TGCGGCGAAC**TTGACA**ATCAGTATTTGCTA**TG**A**TA**A**AAT**CCACCTA |
| P081 | 66700..66745 | - | GTGTCAACAC**TTGA**A**A**TTAACAAAACAGTTAGG**TATA**C**T**ATCATTA |
| P090 | 72039..72084 | + | TGATTGATCA**TTGACA**TAACCACACGGACG**TG**G**TAT**TT**T**TACCTCA |
| P093 | 73059..73104 | + | CTAAAAAGTC**TTGACA**AAAGTGTTATTAATC**TG**A**TAT**T**AT**CGTTCA |
| P105 | 79883..79928 | + | AAGGTCAGTG**TTGAC**TATACCGCTAATAGT**TG**G**TATAA**AATAGGAA |
| P107 | 81153..81198 | + | AACTTTTGTG**TTGAC**TAATAGTATCAGATAATC**TATAAT**AATGGCT |
| P121 | 90197..90242 | + | AAAACAGTTG**TTGACA**AACAGGTAAGCTTTAGA**TAT**T**AT**TATCGGA |
| P144 | 98099..98144 | + | CGACAAACTG**TTGACA**CAACGCTTCAGATTTAG**TAT**C**AT**TGTTTTC |
| P150 | 100005..100050 | + | CACACAGGTG**TTGACA**ACACCAACCCTGTG**TG**AA**ATAAT**ACCTTCA |
| P156 | 101626..101671 | + | AAAAAAGTTC**TTGAC**GGCAAGGATAAATGACAG**TATA**G**T**GACTAAC |
| P162 | 103296..103341 | + | GGCGTTTCTG**TTGACA**CATTACCGCTCCAG**TG**A**TA**GT**AT**CAACACA |
| P164 | 107068..107113 | - | CTATCCTTGG**TTGACA**AGGATCGGCAGTTT**TG**C**TATA**T**T**CTTTTTT |
| P168 | 108112..108157 | - | ATAAAAACCG**TTGACA**TCTGCCAGGGGGTGGGG**TA**AC**AT**TTATCTT |
| P174 | 109634..109679 | - | TTGAAAATAG**TTGACA**TGACCATATAAGACTG**TA**AT**AT**ATAAATCA |
| P185 | 113116..113161 | - | AAAAATAGTG**TTGACA**AGTAAGTCTCAATAAG**TATA**C**T**TAAACACA |
| P186 | 113430..113475 | - | TATTTAAAGC**TTGACA**CCTTGGGATAGTCCGGA**TATA**C**T**AACCACA |
| P192 | 114994..115039 | - | AGACTTTTAA**TTGACA**AGCTAACTTAAATCAG**TATAAT**ACTAAGCA |
| P194 | 115675..115720 | - | ATAAAAATCA**TTGACA**CGCTGCAAAATTTAAG**TA**A**A**C**T**AGACACAA |
| P195 | 116141..116186 | - | AAAATAAGTA**TTGACA**TATAGTCCGACACCAAA**TA**C**A**C**T**AAAAGCA |
| P205 | 118994..119039 | - | CTGTAAAAGA**TTGACA**TCTGAAAACTGCAGTG**TATAAT**ATATGCAT |
| P220 | 125256..125301 | - | AAAATAGTCG**TTGACA**AGAAGTAAGGTTAGATG**TA**AG**AT**ACTCCTC |
| P224 | 126118..126163 | - | TGGAAATGAC**TTGACA**CTGCACCTTGGGAA**TG**C**TAT**T**AT**TGCAGTA |

B. Additional putative promoters discovered using visual inspection

| **Promoter** | **Position** | **Strand** | **Sequence** |
| --- | --- | --- | --- |
| P011 | 6685..6713 | + | **TTGACA**TCTGTTGAGACAAT**TG**A**TA**CG**AT** |
| P020 | 11219..11251 | + | **TTGACA**GACCTAGCGCGGTGGTGGACT**TA**CT**AT** |
| P118 | 88816..88845 | + | **TTGACA**AAGCTTTGTTAAGTTGGT**TATAAT** |
| PtRNA | 62847..62875 | - | **TTGACA**AACTTTGCTTAACAGTG**TA**at**AT** |
| P075 | 63862..63890 | - | **TTGACA**AAAATAATGTTACC**TG**A**TA**ag**AT** |
| P195A | 116444..116472 | - | **TTGACA**GCTCGGACAGTTCTTGT**TAT**tc**T** |
| P231 | 132444..132472 | - | **TTGACA**TTGGTATCCACCGAGTA**TA**c**AAT** |

C. Putative late promoters discovered using MEME


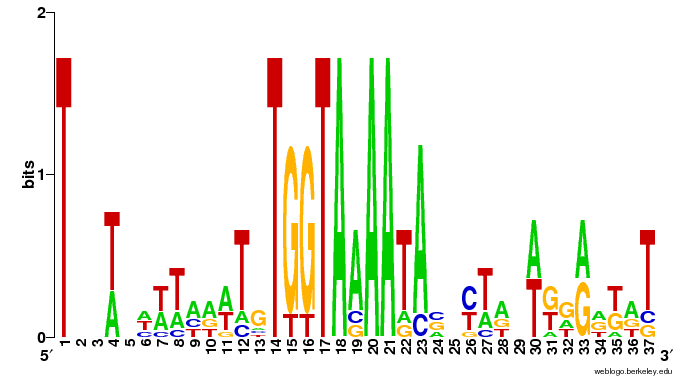


| **Promoter** | **Location** | **Strand** | **Sequence** |
| --- | --- | --- | --- |
| P064 | 57980..58016 | - | TCTTAATTAGATGTGGTACAATAAAGTAATTGGAGAT |
| P056 | 51797..51833 | - | TGATGTATCTTTGTGGTAAAATACATTAGTGAGGGAT |
| P036 | 28605..28641 | - | TTGTTACAAAATTTGGTAAAATACCTTGTAGAGATTT |
| P040 | 34116..34152 | - | TTAACTTAAAATCTGGTAAAATAACTATCATGATTGT |
| P051 | 48276..48312 | - | TGCACCATAATCATGGTAAAATACACAAATATGGTGC |
| P055 | 50899..50935 | - | TATAGATTTTTTGTGGTAGAAGAGCCTGAAGGAGAAG |
| P030 | 22274..22310 | - | TTGTTCTCTAAAGTGTTAAAATAGTCCTAAGGAATTT |
| P048 | 45056..45092 | - | TATTTTATCGGTGTTGTAAAAACGGCTATTTTATGAT |

D. Rho-independent terminators discovered using ARNold and MFOLD

| **Term-inator** | **Position** | **Stand** | **Sequence** | **ΔG (kcal/mol)** |
| --- | --- | --- | --- | --- |
| t003 | 2490..2516 | - | **CCCCGC**TTCG**GCGGGG**ATTTTCTATTT | -16.0 |
| t027 | 13450..13476 | - | **GGGGCC**AAAC**GGCCCC**TTTCTTTTTAT | -13.7 |
| t026 | 13461..13485 | + | **GGGGCC**GTTT**GGCCCC**TTTTTATTT | -13.3 |
| t028 | 19146..19172 | - | **CCGCTCC**TGAC**GGGGCGG**TTTTCTTAT | -12.4 |
| t041 | 34158..34191 | - | **GCCCTCGC**TTCG**GCGGGGGC**TTTTTGTT  TGTAT | -19.8 |
| t049 | 45085..45110 | - | **GGGGGC**TCCG**GCCCCC**TTTATTTTAT | -14.0 |
| t050 | 47469..47508 | - | **GGGCCGGATTCC**TTTTCT**GGGATCTGGCCC**  TTTTTATTAT | -24.2 |
| t052 | 48359..48391 | - | **GCCTCACC**TTCG**GGTGGGGC**TTTTTGC  TTTTAT | -18.3 |
| t060 | 53377..53412 | - | **GGGGAGGGGG**TTCT**CCCCCTCCCC**TTT  TTATTTATT | -24.7 |
| t064ABC | 57949..57975 | - | **GCCCTC**TGAC**GAGGGC**TATTTTGTTAT | -11.5 |
| t099 | 77251..77279 | + | **GGGGAGGC**TTGC**GCCTCCC**TTTTCCT  TTT | -15.3 |
| t109 | 84254..84272 | + | **GGGGC**TTCG**GCCCC**ATTTT | -14.2 |
| t122 | 91702..91724 | + | **GCCCTC**TTCG**GAGGGC**TTTTAT | -14.9 |
| t158 | 102418..102440 | + | **GGGGGGC**TTGG**GCCCCTC**TTTTT | -14.3 |
| t164 | 106573..106598 | - | **GGGGCTT**TAAA**AGGCCCC**TTTCTTTT | -11.9 |
| t177 | 110383..110413 | - | **GGCTATGC**GGTT**GCATGGCC**TTTATT  ATTTT | -13.1 |
| t181 | 111178..111205 | - | **GGCCCCCG**ATCA**TGGGGGCC**TATCTTTT | -16.7 |
| t184 | 111949..111974 | - | **GGCGGCA**TATT**TGCCGCC**TTATTGTT | -14.6 |
| t213 | 121343..121370 | - | **GCCCTGCCC**GAAA**GGGTGGGGC**ATTTTT | -22.3 |
| t232 | 132395..132422 | - | **GGCCCTGCCC**GAAA**GGGTGGGGC**TTTTT | -22.3 |
