# Supplementary material for: The host-range, genomics and proteomics of Escherichia coli O157:H7 bacteriophage rV5
Source: Virol J. 2013 Mar 6;10:76. doi: 10.1186/1743-422X-10-76 (PMC3606486; doi:10.1186/1743-422X-10-76)
Supplement: Additional file 5: Table S5 — MS data on rV5. [file 1743-422X-10-76-S5.html]

D phage
DTASelect v1.9  
/wfs/bfd/22/jcmeng/phage  
/wfs/bfd/scratch/yates/phage\_meng/rv5\_proteins.fasta  
SEQUEST 2.7 in SQT format.  
  
 Jump  to the summary table.  
  
sequest.params modifications:

|  |  |  |
| --- | --- | --- |
| \* | ST | 0.0 |
| # | M | 0.0 |
| @ | C | 0.0 |
| Static | C | 57.0 |

|  |  |
| --- | --- |
| true | Use criteria |
| 1.8 | Minimum +1 XCorr |
| 2.5 | Minimum +2 XCorr |
| 3.5 | Minimum +3 XCorr |
| 0.08 | Minimum DeltCN |
| 1 | Minimum charge state |
| 3 | Maximum charge state |
| 0.0 | Minimum ion proportion |
| 1000 | Maximum Sp rank |
| -1.0 | Minimum Sp score |
| Include | Modified peptide inclusion |
| Any | Tryptic status requirement |
| true | Multiple, ambiguous IDs allowed |
| Ignore | Peptide validation handling |
| XCorr | Purge duplicate peptides by protein |
| false | Include only loci with unique peptide |
| false | Remove subset proteins |
| Ignore | Locus validation handling |
| 0 | Minimum modified peptides per locus |
| 10 | Minimum redundancy for low coverage loci |
| 2 | Minimum peptides per locus |

#### Locus Key:

|  |  |  |  |  |  |  |  |  |
| --- | --- | --- | --- | --- | --- | --- | --- | --- |
| Validation Status | Locus | Sequence Count | Spectrum Count | Sequence Coverage | Length | MolWt | pI | Descriptive Name |

#### Similarity Key:

|  |  |  |
| --- | --- | --- |
| Locus | # of identical peptides | # of differing peptides |

---

|  |  |  |  |  |  |  |  |  |
| --- | --- | --- | --- | --- | --- | --- | --- | --- |
| U | *61* | 49 | 1077 | 85.3% | 129 | 13759 | 6.0 | no description |

| Filename XCorr DeltCN ObsM+H+ CalcM+H+ SpR SpScore Ion% # Sequence  | | | | | | | | | | | |
| --- | --- | --- | --- | --- | --- | --- | --- | --- | --- | --- | --- |
| \* | phage\_03.03522.03522.1 | 3.519 | 0.4684 | 1438.66 | 1439.71 | 1 | 1272.9 | 66.7% | 26 | -.MITAQYSDIVLGK.V | 1 |
| \* | phage\_03.03582.03582.2 | 5.056 | 0.4868 | 1438.93 | 1439.71 | 1 | 2352.6 | 91.7% | 39 | -.MITAQYSDIVLGK.V | 2 |
| \* | phage\_03.02927.02927.2 | 4.2634 | 0.4919 | 1308.41 | 1308.517 | 1 | 1648.3 | 90.9% | 3 | M.ITAQYSDIVLGK.V | 2 |
| \* | phage\_03.02934.02934.1 | 2.3873 | 0.2958 | 1308.63 | 1308.517 | 1 | 542.1 | 50.0% | 1 | M.ITAQYSDIVLGK.V | 1 |
| \* | phage\_03.03729.03729.1 | 2.8709 | 0.3887 | 1195.66 | 1195.358 | 1 | 843.8 | 65.0% | 14 | I.TAQYSDIVLGK.V | 1 |
| \* | phage\_03.03048.03048.1 | 2.5538 | 0.3762 | 1093.67 | 1094.253 | 1 | 888.0 | 66.7% | 1 | T.AQYSDIVLGK.V | 1 |
| \* | phage\_03.02400.02400.2 | 2.9521 | 0.422 | 894.85 | 895.043 | 1 | 706.4 | 85.7% | 1 | Q.YSDIVLGK.V | 2 |
| \* | phage\_03.02405.02405.1 | 2.4106 | 0.1863 | 896.46 | 895.043 | 1 | 889.3 | 71.4% | 3 | Q.YSDIVLGK.V | 1 |
| \* | phage\_03.01884.01884.1 | 1.8462 | 0.2531 | 731.34 | 731.867 | 1 | 632.7 | 75.0% | 1 | Y.SDIVLGK.V | 1 |
| \* | phage\_03.02122.02122.1 | 2.7432 | 0.5357 | 1202.51 | 1203.251 | 1 | 415.0 | 65.0% | 3 | K.VDSSDAGYNFK.E | 1 |
| \* | phage\_05.02053.02053.2 | 3.8539 | 0.5822 | 1203.19 | 1203.251 | 1 | 1416.3 | 85.0% | 4 | K.VDSSDAGYNFK.E | 2 |
| \* | phage\_03.02102.02102.1 | 2.2448 | 0.559 | 1103.45 | 1104.118 | 1 | 486.2 | 61.1% | 1 | V.DSSDAGYNFK.E | 1 |
| \* | phage\_03.02120.02120.1 | 2.3255 | 0.3938 | 990.24 | 989.029 | 1 | 283.9 | 56.2% | 3 | D.SSDAGYNFK.E | 1 |
| \* | phage\_03.02103.02103.1 | 1.8277 | 0.3356 | 814.65 | 814.873 | 1 | 428.5 | 75.0% | 1 | S.DAGYNFK.E | 1 |
| \* | phage\_07.01262.01262.1 | 2.8115 | 0.4703 | 949.5 | 950.085 | 1 | 871.9 | 81.2% | 1 | H.HHAGTVVTK.T | 1 |
| \* | phage\_08.00882.00882.2 | 2.6519 | 0.3796 | 950.35 | 950.085 | 1 | 886.1 | 87.5% | 2 | H.HHAGTVVTK.T | 2 |
| \* | phage\_05.00912.00912.1 | 2.3813 | 0.3838 | 813.41 | 812.944 | 1 | 453.4 | 71.4% | 1 | H.HAGTVVTK.T | 1 |
| \* | phage\_01.02464.02464.1 | 2.5778 | 0.3418 | 1328.39 | 1328.332 | 1 | 306.7 | 50.0% | 4 | K.TGTIANDDGSDVY.G | 1 |
| \* | phage\_03.03792.03792.2 | 6.355 | 0.6958 | 1967.67 | 1968.084 | 1 | 3255.4 | 75.0% | 105 | K.TGTIANDDGSDVYGVLVDR.A | 2 |
| \* | phage\_03.03080.03080.2 | 4.347 | 0.5544 | 1710.69 | 1708.822 | 1 | 924.0 | 56.7% | 1 | T.IANDDGSDVYGVLVDR.A | 2 |
| \* | phage\_03.03179.03179.1 | 2.8861 | 0.5674 | 1594.63 | 1595.663 | 1 | 408.0 | 53.6% | 10 | I.ANDDGSDVYGVLVDR.A | 1 |
| \* | phage\_03.03144.03144.1 | 2.4461 | 0.4442 | 1524.58 | 1524.584 | 1 | 565.3 | 61.5% | 5 | A.NDDGSDVYGVLVDR.A | 1 |
| \* | phage\_02.02700.02700.2 | 4.8271 | 0.5854 | 1410.23 | 1410.48 | 1 | 1889.9 | 87.5% | 10 | N.DDGSDVYGVLVDR.A | 2 |
| \* | phage\_04.02482.02482.1 | 1.9631 | 0.3613 | 780.4 | 778.927 | 4 | 196.4 | 66.7% | 2 | K.VHLAEPL.Q | 1 |
| \* | phage\_05.02368.02368.2 | 3.6142 | 0.4186 | 1321.21 | 1321.519 | 1 | 1037.4 | 77.3% | 73 | K.VHLAEPLQTGQK.Y | 2 |
| \* | phage\_06.02322.02322.1 | 3.4692 | 0.4665 | 1322.68 | 1321.519 | 1 | 671.3 | 63.6% | 37 | K.VHLAEPLQTGQK.Y | 1 |
| \* | phage\_05.02453.02453.2 | 3.1143 | 0.5743 | 1221.91 | 1222.387 | 1 | 744.1 | 80.0% | 7 | V.HLAEPLQTGQK.Y | 2 |
| \* | phage\_05.02339.02339.1 | 2.8476 | 0.525 | 1222.74 | 1222.387 | 1 | 577.6 | 65.0% | 1 | V.HLAEPLQTGQK.Y | 1 |
| \* | phage\_05.02593.02593.1 | 2.5047 | 0.4613 | 1084.2 | 1085.246 | 1 | 356.7 | 55.6% | 17 | H.LAEPLQTGQK.Y | 1 |
| \* | phage\_03.01997.01997.2 | 2.907 | 0.3265 | 1085.17 | 1085.246 | 1 | 578.6 | 77.8% | 1 | H.LAEPLQTGQK.Y | 2 |
| \* | phage\_05.02423.02423.1 | 2.1024 | 0.4608 | 971.61 | 972.086 | 1 | 227.7 | 62.5% | 2 | L.AEPLQTGQK.Y | 1 |
| \* | phage\_06.02280.02280.1 | 2.263 | 0.4146 | 901.28 | 901.007 | 1 | 184.3 | 78.6% | 3 | A.EPLQTGQK.Y | 1 |
| \* | phage\_06.02313.02313.1 | 1.8086 | 0.269 | 771.5 | 771.892 | 1 | 354.8 | 75.0% | 1 | E.PLQTGQK.Y | 1 |
| \* | phage\_12.04271.04271.2 | 2.5427 | 0.3525 | 1244.99 | 1242.507 | 1 | 337.7 | 50.0% | 1 | G.QKYKLVVAVHG.V | 2 |
| \* | phage\_04.03122.03122.1 | 2.7101 | 0.4155 | 1042.49 | 1042.266 | 1 | 567.9 | 72.2% | 8 | K.LVVAVHGVTF.A | 1 |
| \* | phage\_04.03114.03114.2 | 3.6497 | 0.5783 | 1042.97 | 1042.266 | 1 | 1044.6 | 94.4% | 2 | K.LVVAVHGVTF.A | 2 |
| \* | phage\_16.02146.02146.2 | 4.0472 | 0.528 | 1241.07 | 1241.519 | 1 | 1670.9 | 90.9% | 503 | K.LVVAVHGVTFAK.D | 2 |
| \* | phage\_13.03367.03367.1 | 3.5518 | 0.6281 | 1241.99 | 1241.519 | 1 | 1088.0 | 72.7% | 98 | K.LVVAVHGVTFAK.D | 1 |
| \* | phage\_12.00065.00065.1 | 2.8705 | 0.572 | 1028.59 | 1029.227 | 1 | 916.5 | 72.2% | 24 | V.VAVHGVTFAK.D | 1 |
| \* | phage\_11.03775.03775.1 | 2.5639 | 0.6364 | 930.26 | 930.095 | 1 | 674.5 | 68.8% | 9 | V.AVHGVTFAK.D | 1 |
| \* | phage\_03.01187.01187.2 | 2.7198 | 0.1678 | 959.95 | 960.075 | 1 | 433.2 | 83.3% | 3 | K.LLGSNGEAAK.Q | 2 |
| \* | phage\_05.02405.02405.2 | 3.1501 | 0.5315 | 1628.13 | 1628.866 | 1 | 615.8 | 53.3% | 1 | K.LLGSNGEAAKQAVLEK.L | 2 |
| \* | phage\_03.00882.00882.1 | 2.1021 | 0.1708 | 689.38 | 687.814 | 1 | 128.1 | 80.0% | 2 | K.QAVLEK.L | 1 |
| \* | phage\_07.03000.03000.2 | 3.0305 | 0.5337 | 1413.87 | 1413.657 | 1 | 248.0 | 62.5% | 1 | K.QAVLEKLEALGNK.V | 2 |
| \* | phage\_01.02210.02210.1 | 1.82 | 0.3375 | 616.32 | 616.692 | 1 | 488.1 | 80.0% | 1 | K.LEALGN.K | 1 |
| \* | phage\_03.01333.01333.1 | 2.4724 | 0.269 | 746.38 | 744.866 | 1 | 587.8 | 83.3% | 11 | K.LEALGNK.V | 1 |
| \* | phage\_03.02288.02288.2 | 3.3652 | 0.4738 | 1011.55 | 1012.108 | 1 | 1151.2 | 87.5% | 9 | K.VHVQTLGEE.- | 2 |
| \* | phage\_03.02262.02262.1 | 3.0611 | 0.4302 | 1013.46 | 1012.108 | 1 | 645.1 | 75.0% | 19 | K.VHVQTLGEE.- | 1 |
| \* | phage\_01.01984.01984.1 | 1.8688 | 0.448 | 775.35 | 775.834 | 1 | 315.0 | 66.7% | 1 | H.VQTLGEE.- | 1 |

---

|  |  |  |  |  |  |  |  |  |
| --- | --- | --- | --- | --- | --- | --- | --- | --- |
| U | *60* | 88 | 2030 | 83.3% | 336 | 37874 | 6.1 | no description |

| Filename XCorr DeltCN ObsM+H+ CalcM+H+ SpR SpScore Ion% # Sequence  | | | | | | | | | | | |
| --- | --- | --- | --- | --- | --- | --- | --- | --- | --- | --- | --- |
| \* | phage\_16.03936.03936.2 | 6.3228 | 0.6507 | 1960.11 | 1959.25 | 1 | 2401.0 | 76.5% | 48 | R.KDEFGIVDLGATLDLVPR.Q | 2 |
| \* | phage\_07.03737.03737.2 | 5.9969 | 0.6389 | 1830.61 | 1831.076 | 1 | 1525.0 | 75.0% | 79 | K.DEFGIVDLGATLDLVPR.Q | 2 |
| \* | phage\_03.03857.03857.2 | 4.0563 | 0.4233 | 1295.77 | 1296.524 | 1 | 1282.5 | 90.0% | 16 | R.LITGMDLFETR.L | 2 |
| \* | phage\_03.03809.03809.1 | 2.4618 | 0.2071 | 1297.64 | 1296.524 | 1 | 383.2 | 60.0% | 5 | R.LITGMDLFETR.L | 1 |
| \* | phage\_03.02763.02763.1 | 1.9134 | 0.2783 | 1188.68 | 1189.354 | 1 | 140.7 | 45.0% | 1 | R.LGTSTIAQIER.V | 1 |
| \* | phage\_04.02399.02399.2 | 3.8073 | 0.6515 | 1189.01 | 1189.354 | 1 | 1248.2 | 90.0% | 5 | R.LGTSTIAQIER.V | 2 |
| \* | phage\_03.02739.02739.1 | 1.9058 | 0.3664 | 830.61 | 830.96 | 1 | 235.3 | 75.0% | 1 | S.TIAQIER.V | 1 |
| \* | phage\_06.02373.02373.2 | 3.2428 | 0.5353 | 1370.27 | 1370.549 | 1 | 738.2 | 81.8% | 1 | E.RVDEVVTDIPAR.R | 2 |
| \* | phage\_03.02492.02492.2 | 4.3916 | 0.4267 | 1214.11 | 1214.361 | 1 | 2104.3 | 95.0% | 6 | R.VDEVVTDIPAR.R | 2 |
| \* | phage\_03.02452.02452.1 | 3.2077 | 0.2439 | 1215.59 | 1214.361 | 1 | 596.6 | 75.0% | 6 | R.VDEVVTDIPAR.R | 1 |
| \* | phage\_04.02372.02372.2 | 2.8823 | 0.5027 | 1370.41 | 1370.549 | 1 | 475.5 | 59.1% | 1 | R.VDEVVTDIPARR.R | 2 |
| \* | phage\_03.02433.02433.1 | 2.5875 | 0.1844 | 1116.56 | 1115.228 | 1 | 683.4 | 72.2% | 5 | V.DEVVTDIPAR.R | 1 |
| \* | phage\_03.02425.02425.1 | 1.8705 | 0.3993 | 999.57 | 1000.14 | 2 | 313.8 | 68.8% | 1 | D.EVVTDIPAR.R | 1 |
| \* | phage\_02.03445.03445.1 | 2.7236 | 0.2836 | 1190.2 | 1190.384 | 1 | 467.1 | 66.7% | 1 | K.NLNIPFFPLD.K | 1 |
| \* | phage\_04.03542.03542.1 | 3.0999 | 0.3839 | 1317.65 | 1318.558 | 1 | 487.8 | 70.0% | 61 | K.NLNIPFFPLDK.G | 1 |
| \* | phage\_04.00117.00117.2 | 3.8062 | 0.448 | 1317.79 | 1318.558 | 1 | 727.6 | 80.0% | 72 | K.NLNIPFFPLDK.G | 2 |
| \* | phage\_04.03478.03478.2 | 3.2182 | 0.3526 | 1376.11 | 1375.61 | 1 | 413.4 | 63.6% | 1 | K.NLNIPFFPLDKG.I | 2 |
| \* | phage\_04.03483.03483.1 | 2.5946 | 0.3514 | 1203.6 | 1204.455 | 1 | 594.6 | 72.2% | 2 | N.LNIPFFPLDK.G | 1 |
| \* | phage\_04.03489.03489.2 | 2.6167 | 0.3927 | 1204.03 | 1204.455 | 2 | 342.8 | 66.7% | 1 | N.LNIPFFPLDK.G | 2 |
| \* | phage\_04.03514.03514.1 | 2.3056 | 0.0974 | 1092.53 | 1091.295 | 1 | 519.1 | 75.0% | 14 | L.NIPFFPLDK.G | 1 |
| \* | phage\_04.03482.03482.1 | 2.5577 | 0.3561 | 976.55 | 977.191 | 1 | 555.1 | 64.3% | 5 | N.IPFFPLDK.G | 1 |
| \* | phage\_03.04623.04623.1 | 2.423 | 0.2495 | 864.56 | 864.032 | 1 | 597.3 | 83.3% | 42 | I.PFFPLDK.G | 1 |
| \* | phage\_01.02303.02303.1 | 2.3149 | 0.3984 | 888.35 | 888.953 | 1 | 537.5 | 75.0% | 6 | K.GITAADVQN.F | 1 |
| \* | phage\_03.02782.02782.1 | 2.7308 | 0.5177 | 1191.61 | 1192.317 | 1 | 327.7 | 60.0% | 7 | K.GITAADVQNFR.R | 1 |
| \* | phage\_03.02797.02797.2 | 4.7032 | 0.6982 | 1191.77 | 1192.317 | 1 | 1879.3 | 90.0% | 8 | K.GITAADVQNFR.R | 2 |
| \* | phage\_03.02757.02757.1 | 2.25 | 0.47 | 1021.59 | 1022.106 | 1 | 190.4 | 56.2% | 7 | I.TAADVQNFR.R | 1 |
| \* | phage\_03.02779.02779.2 | 2.9317 | 0.508 | 1021.71 | 1022.106 | 1 | 1203.2 | 93.8% | 2 | I.TAADVQNFR.R | 2 |
| \* | phage\_03.02764.02764.1 | 2.3588 | 0.4686 | 920.35 | 921.001 | 1 | 231.4 | 71.4% | 2 | T.AADVQNFR.R | 1 |
| \* | phage\_07.02202.02202.1 | 1.9344 | 0.4874 | 1094.6 | 1095.243 | 1 | 277.9 | 68.8% | 1 | R.RYFTPDAPK.T | 1 |
| \* | phage\_08.02230.02230.2 | 3.28 | 0.4213 | 1095.41 | 1095.243 | 1 | 719.2 | 87.5% | 5 | R.RYFTPDAPK.T | 2 |
| \* | phage\_03.02142.02142.1 | 2.3062 | 0.4177 | 940.45 | 939.056 | 1 | 373.1 | 64.3% | 10 | R.YFTPDAPK.T | 1 |
| \* | phage\_03.01717.01717.1 | 2.0839 | 0.3204 | 917.53 | 918.038 | 1 | 311.2 | 71.4% | 3 | K.TVQDVVTR.V | 1 |
| \* | phage\_03.01742.01742.2 | 2.8021 | 0.4337 | 917.91 | 918.038 | 1 | 761.6 | 92.9% | 4 | K.TVQDVVTR.V | 2 |
| \* | phage\_05.01332.01332.1 | 1.9166 | 0.2226 | 811.52 | 811.916 | 1 | 349.4 | 66.7% | 8 | R.VSHEALR.E | 1 |
| \* | phage\_04.03238.03238.2 | 3.6024 | 0.5744 | 960.77 | 961.192 | 1 | 1056.9 | 93.8% | 5 | K.ALFQAILGK.S | 2 |
| \* | phage\_01.04879.04879.1 | 2.4015 | 0.3744 | 962.11 | 961.192 | 1 | 276.7 | 56.2% | 18 | K.ALFQAILGK.S | 1 |
| \* | phage\_04.02837.02837.1 | 1.9805 | 0.2069 | 889.5 | 890.114 | 1 | 756.1 | 78.6% | 1 | A.LFQAILGK.S | 1 |
| \* | phage\_04.03243.03243.1 | 2.2233 | 0.4092 | 777.27 | 776.954 | 1 | 692.1 | 75.0% | 4 | L.FQAILGK.S | 1 |
| \* | phage\_04.03362.03362.2 | 2.7787 | 0.4932 | 2730.13 | 2732.929 | 1 | 489.4 | 36.4% | 1 | G.KSYAPGDTTCQYDYYTLWGVSQK.S | 2 |
| \* | phage\_01.02993.02993.2 | 2.8734 | 0.6748 | 1704.29 | 1704.71 | 1 | 674.3 | 69.2% | 1 | K.SYAPGDTTCQYDYY.T | 2 |
| \* | phage\_02.03111.03111.2 | 2.5361 | 0.5876 | 2103.85 | 2105.187 | 1 | 540.8 | 56.2% | 1 | K.SYAPGDTTCQYDYYTLW.G | 2 |
| \* | phage\_04.00087.00087.2 | 4.5582 | 0.55 | 2603.03 | 2604.755 | 1 | 559.0 | 45.2% | 157 | K.SYAPGDTTCQYDYYTLWGVSQK.S | 2 |
| \* | phage\_03.03808.03808.3 | 5.6073 | 0.6604 | 2604.68 | 2604.755 | 1 | 1891.4 | 45.2% | 39 | K.SYAPGDTTCQYDYYTLWGVSQK.S | 3 |
| \* | phage\_03.04004.04004.2 | 2.7983 | 0.4991 | 2514.93 | 2517.677 | 1 | 290.6 | 37.5% | 1 | S.YAPGDTTCQYDYYTLWGVSQK.S | 2 |
| \* | phage\_05.03204.03204.2 | 2.6138 | 0.3302 | 2353.39 | 2354.501 | 1 | 835.0 | 47.4% | 1 | Y.APGDTTCQYDYYTLWGVSQK.S | 2 |
| \* | phage\_03.03963.03963.2 | 5.5331 | 0.6551 | 2283.33 | 2283.422 | 1 | 1838.6 | 61.1% | 8 | A.PGDTTCQYDYYTLWGVSQK.S | 2 |
| \* | phage\_03.03889.03889.1 | 2.5806 | 0.4515 | 918.87 | 919.068 | 1 | 636.0 | 78.6% | 4 | Y.TLWGVSQK.S | 1 |
| \* | phage\_01.01874.01874.1 | 1.905 | 0.1263 | 859.33 | 858.967 | 48 | 181.4 | 42.9% | 1 | Q.KSVEIDPA.K | 1 |
| \* | phage\_03.01567.01567.1 | 2.043 | 0.136 | 860.43 | 858.967 | 3 | 518.3 | 64.3% | 6 | K.SVEIDPAK.A | 1 |
| \* | phage\_03.03248.03248.1 | 1.9677 | 0.3435 | 1502.63 | 1503.624 | 2 | 256.7 | 58.3% | 1 | K.ADQDPMEVLEEAR.L | 1 |
| \* | phage\_03.03252.03252.2 | 4.5934 | 0.4796 | 1506.27 | 1503.624 | 1 | 1234.0 | 75.0% | 9 | K.ADQDPMEVLEEAR.L | 2 |
| \* | phage\_03.03259.03259.1 | 1.9204 | 0.3007 | 1188.52 | 1189.326 | 6 | 150.3 | 55.6% | 1 | Q.DPMEVLEEAR.L | 1 |
| \* | phage\_03.03298.03298.1 | 2.6363 | 0.4319 | 1075.42 | 1074.237 | 1 | 471.9 | 68.8% | 4 | D.PMEVLEEAR.L | 1 |
| \* | phage\_03.00965.00965.1 | 1.9227 | 0.2068 | 845.49 | 845.928 | 4 | 177.5 | 75.0% | 1 | M.EVLEEAR.L | 1 |
| \* | phage\_10.03375.03375.1 | 2.5701 | 0.5423 | 1569.78 | 1570.748 | 1 | 564.7 | 57.1% | 4 | R.LHIALQAGDNAAGYR.I | 1 |
| \* | phage\_09.03097.03097.2 | 5.7605 | 0.5932 | 1570.35 | 1570.748 | 1 | 3367.1 | 89.3% | 323 | R.LHIALQAGDNAAGYR.I | 2 |
| \* | phage\_09.03453.03453.1 | 2.5224 | 0.4131 | 1319.63 | 1320.448 | 1 | 266.6 | 50.0% | 9 | H.IALQAGDNAAGYR.I | 1 |
| \* | phage\_03.02327.02327.2 | 3.7076 | 0.6021 | 1319.97 | 1320.448 | 1 | 1305.9 | 79.2% | 1 | H.IALQAGDNAAGYR.I | 2 |
| \* | phage\_10.03427.03427.1 | 2.5382 | 0.3997 | 1207.58 | 1207.288 | 1 | 360.7 | 59.1% | 10 | I.ALQAGDNAAGYR.I | 1 |
| \* | phage\_02.03071.03071.1 | 2.2358 | 0.4055 | 1184.49 | 1183.356 | 1 | 355.0 | 55.6% | 3 | R.IVALCSPEFF.S | 1 |
| \* | phage\_06.02753.02753.2 | 2.7551 | 0.2765 | 1583.53 | 1584.769 | 1 | 1147.8 | 61.5% | 2 | H.HPLVELAYTYYGSA.Q | 2 |
| \* | phage\_03.02297.02297.1 | 2.6359 | 0.4572 | 1284.62 | 1285.399 | 1 | 275.0 | 70.0% | 1 | Y.TYYGSAQEPLR.R | 1 |
| \* | phage\_03.02293.02293.2 | 2.8865 | 0.3806 | 1284.85 | 1285.399 | 1 | 775.1 | 70.0% | 1 | Y.TYYGSAQEPLR.R | 2 |
| \* | phage\_03.01819.01819.2 | 2.8463 | 0.5322 | 1020.85 | 1021.118 | 1 | 442.7 | 75.0% | 1 | Y.YGSAQEPLR.R | 2 |
| \* | phage\_06.02054.02054.2 | 3.2657 | 0.4948 | 1280.39 | 1279.398 | 1 | 869.4 | 81.8% | 4 | R.RLGAGGQDSVYR.V | 2 |
| \* | phage\_03.01833.01833.1 | 2.9101 | 0.4569 | 1122.55 | 1123.211 | 1 | 333.4 | 65.0% | 10 | R.LGAGGQDSVYR.V | 1 |
| \* | phage\_03.01832.01832.2 | 4.1219 | 0.5231 | 1123.15 | 1123.211 | 1 | 1942.2 | 95.0% | 9 | R.LGAGGQDSVYR.V | 2 |
| \* | phage\_03.01872.01872.1 | 2.2443 | 0.5437 | 1009.54 | 1010.051 | 1 | 181.9 | 50.0% | 5 | L.GAGGQDSVYR.V | 1 |
| \* | phage\_03.01880.01880.1 | 2.0265 | 0.4604 | 952.52 | 952.999 | 1 | 228.2 | 62.5% | 2 | G.AGGQDSVYR.V | 1 |
| \* | phage\_04.03798.03798.1 | 3.643 | 0.5429 | 1503.73 | 1504.723 | 1 | 502.2 | 57.7% | 61 | K.GITFIEDISGNIPK.K | 1 |
| \* | phage\_03.04464.04464.2 | 5.0817 | 0.5472 | 1507.17 | 1504.723 | 1 | 1250.3 | 76.9% | 152 | K.GITFIEDISGNIPK.K | 2 |
| \* | phage\_04.04142.04142.1 | 2.6961 | 0.189 | 1333.46 | 1334.512 | 3 | 356.4 | 54.5% | 20 | I.TFIEDISGNIPK.K | 1 |
| \* | phage\_04.03963.03963.2 | 4.6806 | 0.6052 | 1333.85 | 1334.512 | 1 | 1309.9 | 81.8% | 9 | I.TFIEDISGNIPK.K | 2 |
| \* | phage\_04.04150.04150.2 | 2.7452 | 0.2856 | 1233.57 | 1233.407 | 2 | 570.8 | 65.0% | 1 | T.FIEDISGNIPK.K | 2 |
| \* | phage\_04.04070.04070.1 | 3.356 | 0.3104 | 1234.39 | 1233.407 | 5 | 483.0 | 55.0% | 34 | T.FIEDISGNIPK.K | 1 |
| \* | phage\_05.00014.00014.1 | 2.3572 | 0.1173 | 1085.54 | 1086.23 | 1 | 666.9 | 66.7% | 17 | F.IEDISGNIPK.K | 1 |
| \* | phage\_05.03260.03260.1 | 2.0346 | 0.1342 | 972.57 | 973.071 | 1 | 417.1 | 62.5% | 3 | I.EDISGNIPK.K | 1 |
| \* | phage\_04.03989.03989.1 | 1.8772 | 0.1457 | 843.59 | 843.955 | 3 | 659.6 | 78.6% | 1 | E.DISGNIPK.K | 1 |
| \* | phage\_04.03563.03563.2 | 3.9034 | 0.6286 | 1542.65 | 1543.882 | 1 | 614.5 | 83.3% | 2 | R.ILPMGIDQMFQLH.F | 2 |
| \* | phage\_03.03388.03388.2 | 3.8879 | 0.5076 | 2344.79 | 2344.499 | 1 | 1002.3 | 52.5% | 1 | F.QLHFAPADDVNEANTPAQELY.M | 2 |
| \* | phage\_04.02814.02814.2 | 3.8068 | 0.6302 | 2101.91 | 2103.209 | 1 | 1176.8 | 58.3% | 1 | L.HFAPADDVNEANTPAQELY.M | 2 |
| \* | phage\_04.03367.03367.2 | 4.8194 | 0.526 | 2573.69 | 2574.824 | 1 | 1202.3 | 57.1% | 6 | H.FAPADDVNEANTPAQELYMWYK.H | 2 |
| \* | phage\_07.01412.01412.1 | 1.8193 | 0.4965 | 746.48 | 746.844 | 1 | 239.9 | 80.0% | 2 | K.HSAYLR.E | 1 |
| \* | phage\_12.03801.03801.3 | 4.5412 | 0.6107 | 2117.12 | 2117.466 | 1 | 571.9 | 40.3% | 95 | K.IESETSMLAVNTRPELVVK.A | 3 |
| \* | phage\_13.03756.03756.2 | 5.1067 | 0.5563 | 2120.23 | 2117.466 | 1 | 1205.7 | 58.3% | 527 | K.IESETSMLAVNTRPELVVK.A | 2 |
| \* | phage\_11.04170.04170.2 | 2.6792 | 0.4709 | 1875.47 | 1875.191 | 1 | 623.2 | 46.9% | 5 | E.SETSMLAVNTRPELVVK.A | 2 |
| \* | phage\_05.02382.02382.2 | 2.9058 | 0.4729 | 1226.53 | 1226.462 | 1 | 630.8 | 70.0% | 2 | L.AVNTRPELVVK.A | 2 |
| \* | phage\_07.02127.02127.2 | 2.9015 | 0.4992 | 942.19 | 942.147 | 1 | 683.3 | 85.7% | 5 | N.TRPELVVK.A | 2 |

---

|  |  |  |  |  |  |  |  |  |
| --- | --- | --- | --- | --- | --- | --- | --- | --- |
| U | *64* | 17 | 23 | 36.3% | 518 | 57130 | 6.5 | no description |

| Filename XCorr DeltCN ObsM+H+ CalcM+H+ SpR SpScore Ion% # Sequence  | | | | | | | | | | | |
| --- | --- | --- | --- | --- | --- | --- | --- | --- | --- | --- | --- |
| \* | phage\_06.00769.00769.2 | 2.6916 | 0.4287 | 1181.13 | 1181.253 | 1 | 631.2 | 72.7% | 1 | K.SGNAGAPTHNVR.M | 2 |
| \* | phage\_03.02244.02244.2 | 3.6188 | 0.6129 | 1282.29 | 1282.356 | 1 | 1295.6 | 81.8% | 1 | K.CDSTVATALDTK.Y | 2 |
| \* | phage\_04.01692.01692.2 | 3.3505 | 0.447 | 1057.73 | 1058.183 | 1 | 845.3 | 87.5% | 1 | R.NLANQQTLR.D | 2 |
| \* | phage\_05.03388.03388.2 | 2.8345 | 0.5428 | 1711.53 | 1711.868 | 1 | 647.4 | 50.0% | 1 | R.SAATFNEYGFSLFEK.V | 2 |
| \* | phage\_09.02453.02453.2 | 2.907 | 0.4857 | 1245.87 | 1246.455 | 1 | 706.0 | 75.0% | 1 | K.IAFRPQASLSR.S | 2 |
| \* | phage\_04.02934.02934.2 | 5.2217 | 0.6423 | 2108.83 | 2110.334 | 1 | 1671.5 | 66.7% | 3 | R.TLTGIYQSPNAFLNTQNAR.W | 2 |
| \* | phage\_04.02770.02770.2 | 3.472 | 0.3372 | 1128.85 | 1129.362 | 1 | 1129.6 | 90.0% | 3 | R.WAGPLAAMPSK.G | 2 |
| \* | phage\_03.03305.03305.2 | 3.7541 | 0.5056 | 1362.13 | 1362.568 | 1 | 1228.6 | 81.8% | 1 | K.GFNEPEIFIPAK.K | 2 |
| \* | phage\_04.02243.02243.1 | 2.0302 | 0.2776 | 898.52 | 899.078 | 1 | 429.4 | 71.4% | 1 | K.IPSNILNK.A | 1 |
| \* | phage\_04.02248.02248.2 | 3.1458 | 0.3371 | 899.03 | 899.078 | 2 | 611.0 | 92.9% | 1 | K.IPSNILNK.A | 2 |
| \* | phage\_03.02065.02065.2 | 2.7052 | 0.5423 | 1177.15 | 1177.274 | 1 | 673.7 | 80.0% | 1 | K.ASIDPNSMEGR.M | 2 |
| \* | phage\_04.03430.03430.2 | 4.2943 | 0.6295 | 2261.37 | 2261.453 | 1 | 675.9 | 40.5% | 2 | R.FGAGFINLGNDNVGSFSLSESK.Q | 2 |
| \* | phage\_03.03550.03550.2 | 3.9984 | 0.5327 | 1281.17 | 1279.433 | 1 | 1560.4 | 85.0% | 1 | R.DIDIITEAFNK.D | 2 |
| \* | phage\_03.03417.03417.2 | 2.6598 | 0.3192 | 1383.95 | 1385.69 | 5 | 277.9 | 45.5% | 1 | A.FNKDLIPQLLAL.N | 2 |
| \* | phage\_04.02468.02468.2 | 3.1574 | 0.5357 | 917.77 | 918.124 | 1 | 1430.5 | 93.8% | 1 | R.IGAVGYLPK.T | 2 |
| \* | phage\_03.01167.01167.1 | 2.4296 | 0.4845 | 742.59 | 742.894 | 1 | 468.2 | 75.0% | 2 | K.TPAVINK.I | 1 |
| \* | phage\_03.02912.02912.2 | 3.3941 | 0.2446 | 1135.19 | 1135.262 | 1 | 1115.2 | 83.3% | 1 | K.ILEVGGFDER.F | 2 |

---

|  |  |  |  |  |  |  |  |  |
| --- | --- | --- | --- | --- | --- | --- | --- | --- |
| U | *53* | 10 | 15 | 31.9% | 458 | 50272 | 5.3 | no description |

| Filename XCorr DeltCN ObsM+H+ CalcM+H+ SpR SpScore Ion% # Sequence  | | | | | | | | | | | |
| --- | --- | --- | --- | --- | --- | --- | --- | --- | --- | --- | --- |
| \* | phage\_05.03257.03257.2 | 2.5286 | 0.5462 | 1639.77 | 1639.956 | 1 | 433.4 | 50.0% | 1 | K.AFGGVFPPQYVMIGR.Q | 2 |
| \* | phage\_09.02973.02973.2 | 2.5837 | 0.1895 | 2239.93 | 2239.589 | 4 | 232.1 | 28.6% | 2 | E.KTTVDFKGIAAAADTDVVMTIA.K | 2 |
| \* | phage\_04.02120.02120.2 | 4.1566 | 0.4579 | 1487.85 | 1486.665 | 1 | 1480.7 | 69.2% | 1 | K.NIANAEKPSTVVDK.I | 2 |
| \* | phage\_06.02433.02433.2 | 4.0427 | 0.6235 | 1420.29 | 1420.562 | 1 | 1356.9 | 83.3% | 3 | K.LHVYSTAEDVTGK.D | 2 |
| \* | phage\_06.03305.03305.2 | 4.4817 | 0.5172 | 2592.61 | 2592.84 | 1 | 550.7 | 39.6% | 1 | K.DFPEGGIIGAMASNDPSYGDSLHLK.Q | 2 |
| \* | phage\_05.02353.02353.2 | 3.31 | 0.5902 | 1503.83 | 1504.596 | 1 | 643.1 | 61.5% | 1 | M.ASNDPSYGDSLHLK.Q | 2 |
| \* | phage\_03.02904.02904.2 | 3.5802 | 0.494 | 1402.15 | 1401.664 | 1 | 1244.1 | 79.2% | 1 | K.SVLMNNPINTGIK.N | 2 |
| \* | phage\_02.03350.03350.2 | 3.4262 | 0.4175 | 2653.93 | 2653.948 | 1 | 778.8 | 41.3% | 3 | K.NGAILTGFDEDNNVFYDPIITVPK.R | 2 |
| \* | phage\_03.02295.02295.2 | 2.642 | 0.5582 | 1183.85 | 1184.338 | 1 | 695.9 | 80.0% | 1 | R.AEIPTNQLAAR.V | 2 |
| \* | phage\_01.02244.02244.1 | 1.8922 | 0.4203 | 923.34 | 924.014 | 1 | 419.3 | 68.8% | 1 | K.STNAVAMTE.- | 1 |

---

|  |  |  |  |  |  |  |  |  |
| --- | --- | --- | --- | --- | --- | --- | --- | --- |
| U | *52* | 3 | 4 | 16.6% | 157 | 17183 | 4.8 | no description |

| Filename XCorr DeltCN ObsM+H+ CalcM+H+ SpR SpScore Ion% # Sequence  | | | | | | | | | | | |
| --- | --- | --- | --- | --- | --- | --- | --- | --- | --- | --- | --- |
| \* | phage\_03.01644.01644.1 | 1.925 | 0.3317 | 909.5 | 910.015 | 1 | 748.6 | 68.8% | 1 | R.VTGFAADTK.I | 1 |
| \* | phage\_02.03605.03605.2 | 3.7878 | 0.5991 | 1603.97 | 1604.844 | 1 | 1199.5 | 65.6% | 2 | R.DNITGALTGLAGFAGII.- | 2 |
| \* | phage\_02.03617.03617.1 | 2.5016 | 0.3383 | 1604.53 | 1604.844 | 2 | 235.5 | 37.5% | 1 | R.DNITGALTGLAGFAGII.- | 1 |

---

|  |  |  |  |  |  |  |  |  |
| --- | --- | --- | --- | --- | --- | --- | --- | --- |
| U | *133* | 2 | 2 | 15.9% | 151 | 17627 | 8.7 | no description |

| Filename XCorr DeltCN ObsM+H+ CalcM+H+ SpR SpScore Ion% # Sequence  | | | | | | | | | | | |
| --- | --- | --- | --- | --- | --- | --- | --- | --- | --- | --- | --- |
| \* | phage\_05.03352.03352.2 | 2.5424 | 0.3244 | 2355.37 | 2354.461 | 1 | 364.5 | 33.3% | 1 | P.CDQREYVFDSVTERDHPSL.M | 2 |
| \* | phage\_02.02189.02189.1 | 1.9018 | 0.136 | 584.6 | 584.82 | 5 | 222.1 | 75.0% | 1 | Q.LLLIL.H | 1 |

---

|  |  |  |  |  |  |  |  |  |
| --- | --- | --- | --- | --- | --- | --- | --- | --- |
| U | *79* | 2 | 2 | 15.5% | 116 | 12803 | 9.4 | no description |

| Filename XCorr DeltCN ObsM+H+ CalcM+H+ SpR SpScore Ion% # Sequence  | | | | | | | | | | | |
| --- | --- | --- | --- | --- | --- | --- | --- | --- | --- | --- | --- |
| \* | phage\_01.02943.02943.1 | 1.8577 | 0.089 | 1332.53 | 1329.709 | 20 | 175.6 | 40.0% | 1 | D.KLLYLKVPLEI.L | 1 |
| \* | phage\_03.03073.03073.1 | 2.0752 | 0.1755 | 961.52 | 961.069 | 79 | 220.3 | 50.0% | 1 | Y.DKIVYCY.L | 1 |

---

|  |  |  |  |  |  |  |  |  |
| --- | --- | --- | --- | --- | --- | --- | --- | --- |
| U | *152* | 2 | 2 | 12.8% | 109 | 12341 | 4.6 | no description |

| Filename XCorr DeltCN ObsM+H+ CalcM+H+ SpR SpScore Ion% # Sequence  | | | | | | | | | | | |
| --- | --- | --- | --- | --- | --- | --- | --- | --- | --- | --- | --- |
| \* | phage\_04.02383.02383.1 | 1.8175 | 0.2424 | 816.64 | 815.991 | 4 | 133.1 | 58.3% | 1 | L.LKARAEK.A | 1 |
| \* | phage\_03.00778.00778.1 | 1.8929 | 0.2124 | 910.29 | 911.101 | 1 | 586.6 | 83.3% | 1 | K.YLMEILE.I | 1 |

---

|  |  |  |  |  |  |  |  |  |
| --- | --- | --- | --- | --- | --- | --- | --- | --- |
| U | *82* | 2 | 14 | 12.5% | 311 | 36090 | 6.4 | no description |

| Filename XCorr DeltCN ObsM+H+ CalcM+H+ SpR SpScore Ion% # Sequence  | | | | | | | | | | | |
| --- | --- | --- | --- | --- | --- | --- | --- | --- | --- | --- | --- |
| \* | phage\_06.03823.03823.2 | 2.9146 | 0.1114 | 2211.27 | 2212.523 | 1 | 402.0 | 32.5% | 12 | R.YNDSGIMLPEAALDGIAKYLG.A | 2 |
| \* | phage\_04.00054.00054.2 | 2.5555 | 0.191 | 2367.77 | 2369.522 | 2 | 309.1 | 29.4% | 2 | I.RSNYTEDVWNCLLEQERR.E | 2 |

---

|  |  |  |  |  |  |  |  |  |
| --- | --- | --- | --- | --- | --- | --- | --- | --- |
| U | *34* | 4 | 18 | 10.3% | 690 | 79101 | 5.2 | no description |

| Filename XCorr DeltCN ObsM+H+ CalcM+H+ SpR SpScore Ion% # Sequence  | | | | | | | | | | | |
| --- | --- | --- | --- | --- | --- | --- | --- | --- | --- | --- | --- |
| \* | phage\_04.03037.03037.2 | 2.9978 | 0.3764 | 2145.27 | 2146.484 | 1 | 612.9 | 44.1% | 1 | L.DLLQPDTGWKMMQLNNIQ.V | 2 |
| \* | phage\_07.00205.00205.2 | 2.584 | 0.1637 | 2229.69 | 2229.631 | 1 | 361.6 | 34.2% | 1 | N.VSPLRVLNNIKRLGYTGTIN.H | 2 |
| \* | phage\_05.03455.03455.2 | 2.5633 | 0.0845 | 1340.45 | 1341.643 | 16 | 233.6 | 41.7% | 1 | C.RGKLGARIAAISK.A | 2 |
| \* | phage\_08.00219.00219.2 | 3.1005 | 0.1879 | 2225.89 | 2226.624 | 1 | 562.1 | 39.5% | 15 | S.VNIPIVKLGWQPSEIQYLAG.F | 2 |

---

|  |  |  |  |  |  |  |  |  |
| --- | --- | --- | --- | --- | --- | --- | --- | --- |
| U | *237* | 3 | 12 | 9.9% | 456 | 51380 | 7.6 | no description |

| Filename XCorr DeltCN ObsM+H+ CalcM+H+ SpR SpScore Ion% # Sequence  | | | | | | | | | | | |
| --- | --- | --- | --- | --- | --- | --- | --- | --- | --- | --- | --- |
| \* | phage\_02.03730.03730.2 | 3.2894 | 0.1105 | 1302.01 | 1301.528 | 1 | 722.6 | 72.7% | 1 | K.NDKPFGGIQVIL.F | 2 |
| \* | phage\_08.03393.03393.2 | 3.0772 | 0.1368 | 1574.61 | 1571.733 | 1 | 672.5 | 54.2% | 10 | L.HNIALIDQFRQES.I | 2 |
| \* | phage\_04.03910.03910.2 | 2.6538 | 0.1232 | 2367.97 | 2368.692 | 8 | 173.1 | 23.7% | 1 | I.VDPRVVEFYNTTFPDLDKIV.R | 2 |

---

|  |  |  |  |  |  |  |  |  |
| --- | --- | --- | --- | --- | --- | --- | --- | --- |
| U | *41* | 8 | 9 | 8.1% | 1272 | 139488 | 5.8 | no description |

| Filename XCorr DeltCN ObsM+H+ CalcM+H+ SpR SpScore Ion% # Sequence  | | | | | | | | | | | |
| --- | --- | --- | --- | --- | --- | --- | --- | --- | --- | --- | --- |
| \* | phage\_05.02124.02124.2 | 3.2104 | 0.5692 | 985.07 | 985.085 | 1 | 1090.3 | 83.3% | 1 | K.SASGILHGDK.Y | 2 |
| \* | phage\_07.02357.02357.2 | 3.8806 | 0.6252 | 1424.25 | 1424.513 | 1 | 1233.1 | 77.3% | 1 | K.YNTDGVLEHGYR.I | 2 |
| \* | phage\_04.01782.01782.1 | 1.9152 | 0.3583 | 754.45 | 754.948 | 1 | 571.2 | 71.4% | 1 | K.VPIAAGVK.D | 1 |
| \* | phage\_03.01628.01628.1 | 1.9254 | 0.2955 | 976.46 | 976.118 | 4 | 107.4 | 56.2% | 1 | T.GTDITIVTR.S | 1 |
| \* | phage\_04.02719.02719.2 | 3.0763 | 0.4538 | 1028.99 | 1029.286 | 1 | 959.1 | 72.2% | 1 | K.ALLGMVAPTR.G | 2 |
| \* | phage\_03.02294.02294.2 | 4.693 | 0.5641 | 1866.27 | 1866.936 | 1 | 1369.6 | 68.8% | 1 | R.EQDYQVGAVTDNQDGVK.F | 2 |
| \* | phage\_09.03920.03920.2 | 3.0295 | 0.2714 | 2234.23 | 2234.34 | 1 | 369.0 | 37.5% | 2 | L.FSNAGFQAASEGTEDLINNTF.T | 2 |
| \* | phage\_03.02874.02874.2 | 3.6725 | 0.6613 | 1689.45 | 1689.86 | 1 | 872.2 | 70.0% | 1 | R.YGGTTPEGEIPTPELK.V | 2 |

---

|  |  |  |  |  |  |  |  |  |
| --- | --- | --- | --- | --- | --- | --- | --- | --- |
| U | *62* | 2 | 2 | 7.7% | 325 | 36639 | 4.8 | no description |

| Filename XCorr DeltCN ObsM+H+ CalcM+H+ SpR SpScore Ion% # Sequence  | | | | | | | | | | | |
| --- | --- | --- | --- | --- | --- | --- | --- | --- | --- | --- | --- |
| \* | phage\_04.02763.02763.2 | 2.8358 | 0.4855 | 1830.93 | 1831.815 | 1 | 595.7 | 53.6% | 1 | K.EHPDWEWAQADAESC.I | 2 |
| \* | phage\_03.02998.02998.1 | 1.9551 | 0.0847 | 1250.38 | 1251.509 | 12 | 216.3 | 55.6% | 1 | T.KTKEYLDLLK.Q | 1 |

---

|  |  |  |  |  |  |  |  |  |
| --- | --- | --- | --- | --- | --- | --- | --- | --- |
| U | *235* | 2 | 44 | 7.5% | 441 | 49967 | 6.8 | no description |

| Filename XCorr DeltCN ObsM+H+ CalcM+H+ SpR SpScore Ion% # Sequence  | | | | | | | | | | | |
| --- | --- | --- | --- | --- | --- | --- | --- | --- | --- | --- | --- |
| \* | phage\_07.03180.03180.2 | 3.714 | 0.3007 | 2216.65 | 2215.439 | 1 | 500.0 | 41.2% | 43 | V.KCVYIDPPFNTGRAFEHY.D | 2 |
| \* | phage\_03.03112.03112.1 | 1.8179 | 0.1603 | 1456.69 | 1455.518 | 158 | 19.6 | 25.0% | 1 | R.EGDLVLDSFGGSGTT.A | 1 |

---

|  |  |  |  |  |  |  |  |  |
| --- | --- | --- | --- | --- | --- | --- | --- | --- |
| U | *106* | 2 | 2 | 5.2% | 327 | 37636 | 6.6 | no description |

| Filename XCorr DeltCN ObsM+H+ CalcM+H+ SpR SpScore Ion% # Sequence  | | | | | | | | | | | |
| --- | --- | --- | --- | --- | --- | --- | --- | --- | --- | --- | --- |
| \* | phage\_02.03435.03435.1 | 1.8561 | 0.1327 | 1005.54 | 1005.166 | 2 | 416.6 | 68.8% | 1 | K.LCTAKIIAD.S | 1 |
| \* | phage\_04.02974.02974.1 | 1.866 | 0.2003 | 993.62 | 996.12 | 10 | 245.8 | 57.1% | 1 | D.MELTPEEF.W | 1 |

---

|  |  |  |  |  |  |  |  |  |
| --- | --- | --- | --- | --- | --- | --- | --- | --- |
| U | *37* | 4 | 5 | 4.2% | 1279 | 140069 | 5.5 | no description |

| Filename XCorr DeltCN ObsM+H+ CalcM+H+ SpR SpScore Ion% # Sequence  | | | | | | | | | | | |
| --- | --- | --- | --- | --- | --- | --- | --- | --- | --- | --- | --- |
| \* | phage\_04.03660.03660.2 | 3.2851 | 0.5999 | 2130.21 | 2131.346 | 1 | 799.0 | 52.9% | 2 | K.GYSETDFPSVFVPEWIEK.D | 2 |
| \* | phage\_03.03968.03968.2 | 2.6099 | 0.2142 | 2204.77 | 2203.576 | 1 | 465.9 | 41.2% | 1 | I.IEVTCNMRGFKFKPGRYV.T | 2 |
| \* | phage\_03.01570.01570.1 | 2.062 | 0.2323 | 803.47 | 802.903 | 1 | 358.9 | 57.1% | 1 | Q.AIDALDGK.L | 1 |
| \* | phage\_04.03260.03260.1 | 1.8552 | 0.0838 | 964.39 | 964.038 | 7 | 354.7 | 50.0% | 1 | I.QLGMEGSGGQ.V | 1 |

---

|  |  |  |  |  |  |  |  |  |
| --- | --- | --- | --- | --- | --- | --- | --- | --- |
| U | *1* | 2 | 2 | 3.2% | 660 | 75811 | 8.2 | no description |

| Filename XCorr DeltCN ObsM+H+ CalcM+H+ SpR SpScore Ion% # Sequence  | | | | | | | | | | | |
| --- | --- | --- | --- | --- | --- | --- | --- | --- | --- | --- | --- |
| \* | phage\_03.03053.03053.1 | 2.1316 | 0.202 | 1456.55 | 1457.681 | 1 | 60.7 | 41.7% | 1 | A.PFSMVDTFDVISV.H | 1 |
| \* | phage\_04.02260.02260.1 | 2.1007 | 0.1445 | 900.57 | 900.167 | 8 | 272.8 | 57.1% | 1 | Q.LGKIIPMQ.D | 1 |

---

|  |  |  |  |  |  |  |  |  |
| --- | --- | --- | --- | --- | --- | --- | --- | --- |
| U | *27* | 3 | 9 | 3.1% | 1878 | 208334 | 6.7 | no description |

| Filename XCorr DeltCN ObsM+H+ CalcM+H+ SpR SpScore Ion% # Sequence  | | | | | | | | | | | |
| --- | --- | --- | --- | --- | --- | --- | --- | --- | --- | --- | --- |
| \* | phage\_04.03285.03285.2 | 2.8891 | 0.404 | 2589.57 | 2590.784 | 1 | 228.8 | 31.8% | 3 | N.LCNDIGAQSANNSPRANFIHYRA.L | 2 |
| \* | phage\_04.04205.04205.2 | 3.1986 | 0.1061 | 1508.43 | 1506.887 | 9 | 271.9 | 45.8% | 4 | N.IYIGDTVKMKLKP.Q | 2 |
| \* | phage\_06.03573.03573.2 | 2.6517 | 0.1713 | 2212.41 | 2214.304 | 14 | 204.2 | 23.8% | 2 | R.YADGTTNNIGITLGGTSEDNSL.K | 2 |

---

|  |  |  |  |  |  |  |  |  |
| --- | --- | --- | --- | --- | --- | --- | --- | --- |
| U | *228* | 2 | 7 | 2.7% | 775 | 89191 | 8.6 | no description |

| Filename XCorr DeltCN ObsM+H+ CalcM+H+ SpR SpScore Ion% # Sequence  | | | | | | | | | | | |
| --- | --- | --- | --- | --- | --- | --- | --- | --- | --- | --- | --- |
| \* | phage\_09.03254.03254.2 | 3.2386 | 0.3633 | 1379.43 | 1379.556 | 2 | 364.1 | 45.5% | 6 | N.QDDIKRYLISAG.W | 2 |
| \* | phage\_01.02948.02948.1 | 1.9675 | 0.0899 | 1058.24 | 1057.235 | 1 | 347.5 | 62.5% | 1 | R.RSVLDPIKE.D | 1 |

|  |  |  |  |
| --- | --- | --- | --- |
|  | Proteins | Peptide IDs | Copies |
| Unfiltered | 232 | 75804 | 78255 |
| Redundant | 19 | 207 | 3279 |
| Nonredundant | 19 | 207 | 3279 |

  
/wfs/bfd/22/jcmeng/phage
